# Insights gained from single-cell sequencing analysis of ischemic stroke

**DOI:** 10.3389/fcell.2026.1784660

**Published:** 2026-04-21

**Authors:** Yanqin Mei, Teng Guan, Longsheng Fu, Jiming Kong, Yanni Lv

**Affiliations:** 1 Department of Pharmacy, The First Affiliated Hospital, Jiangxi Medical College, Nanchang University, Nanchang, China; 2 School of Pharmacy, Jiangxi Medical College, Nanchang University, Nanchang, China; 3 Max Rady College of Medicine, Department of Human Anatomy and Cell Science, University of Manitoba, Winnipeg, Canada; 4 Key Laboratory of Rare Neurological Diseases of Jiangxi Provincial Health Commission, The First Affiliated Hospital, Jiangxi Medical College, Nanchang University, Nanchang, China; 5 Jiangxi Key Laboratory of Neurological Diseases, The First Affiliated Hospital, Jiangxi Medical College, Nanchang University, Nanchang, China

**Keywords:** applied application, cell heterogeneity, cell subcluster, immune response, ischemic stroke, single-cell RNA sequencing

## Abstract

Ischemic stroke represents a significant global health hazard, causing substantial morbidity, mortality, and long-term disability worldwide. The brain’s complexity necessitates targeting ischemic stroke dysfunction syndrome. Single-cell RNA sequencing (scRNA-seq) offers unbiased, high-resolution analysis of cell heterogeneity, revealing cellular markers and types. Limited therapeutic options and drug discovery hinder stroke treatment, but scRNA-seq provides insights into new targets and biomarkers. Since 2019, over 50 studies on scRNA-seq in ischemic stroke have been published. This review summarizes ischemic stroke research based on scRNA-seq technology, detailing stroke features from various perspectives. We tabulated 22 brain and 7 blood cell types post-stroke. 13 cell types that frequently reported in ischemic stroke and undergone extensive heterogeneity studies were summarized. A summary was given of investigations into subcluster categorization, function-related subclusters, and newly discovered subclusters from scRNA-seq. Immune cell or non-immune cellular interactions and their regulatory influences were also explored. Additionally, the review covered age-related issues, advantages and disadvantages, technological innovations, and scope in ischemic stroke. Despite the moniker “single-cell,” the analysis transcends individual entities, acknowledging the systemic variability of cellular types. Single-cell sequencing technologies, by their nature, interrogate entire tissues, distilling information from thousands of cells into a comprehensive average. Looking ahead, there is anticipation for broader scRNA-seq application in ischemic stroke research, promising deeper understanding of cellular complexities.

## Graphical Abstract

**Figure:**
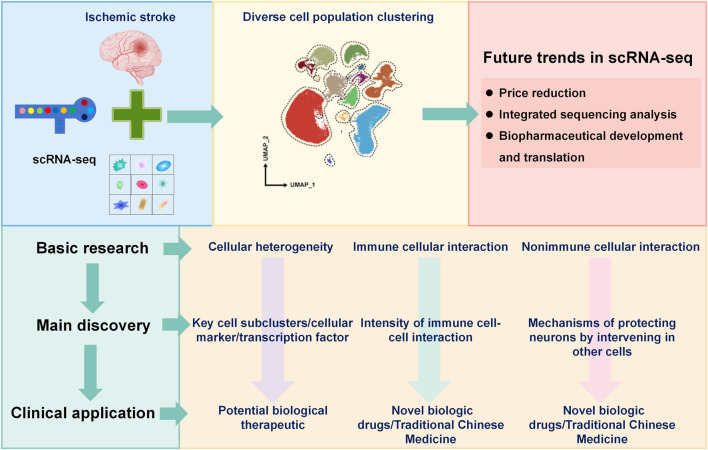

## 1 Introduction

The brain is the body’s most vital organ, if it stops working, death soon follows. The structure and function of brain are the most difficult problems in natural science research. As Cajal, the founder of modern brain science, said, “As long as the mysteries of the brain are not revealed, the universe will remain a mystery.” The map came out in 2021 from the scientific magazine《Nature》, which issued a so far most complete map of brain cells released from BRAIN Initiative Cell Census Network (BRAIN Initiative Cell Census Network BICCN, 2021; Muñoz-Castañeda et al., 2021). Thus, the number of identified brain cell types has increased fivefold, and the identification of cell subtypes rises to a higher step than before (BRAIN Initiative Cell Census Network BICCN, 2021; Muñoz-Castañeda et al., 2021). Single-cell RNA sequencing (scRNA-seq) allows for unbiased, high-resolution analysis of cell heterogeneity, aiding in the identification of cellular markers and types via dimensionality reduction clustering (Qiu et al., 2022). This technological advancement has empowered researchers to perform high-resolution, in-depth characterization of gene expression profiles and cell-cell interaction networks in brain research.

Cerebral ischemia represents a disease marked by elevated incidence, mortality, and disability rates, positioning it as a significant chronic condition that necessitates worldwide prevention and treatment initiatives. Scientists have been collectively and wholeheartedly committed to both clinical and fundamental research on cerebral ischemia, with a particular emphasis on the realm of basic research. Stroke causes widespread cellular changes, but traditional methods only validated limited cell types related to ischemic stroke. The results of traditional sequencing techniques (as traditional experimental method: Western blot, real-time PCR, ELISA) are generally displayed with the average mean of thousands of cells (as the expression change of protein, even the differential expression within gene-omics, transcription-omics, and protein-omics level) (Ren et al., 2021). Currently, stroke treatment options are limited, but scRNA-seq may facilitate the identification of novel therapeutic targets and biomarkers for stroke reduction (Qiu et al., 2022). Scientists use scRNA-seq to understand cell-to-cell communication, focusing on subclusters and states. It also offers a new view of brain cellular differentiation via pseudo-time analysis. Latest scRNA-seq technologies like genomics, proteomics, and metabonomics promise deeper insights into brain science. scRNA-seq technology start a bit late in ischemic stroke. There are about more than 50 basic researches published since 2019. In this review, we collected the relevant literature using the keywords “scRNA-seq” and “Ischemic Stroke”, along with keywords adjusted through MeSH (Medical Subject Headings) calibration. Meanwhile, we collect only basic research to the review using primary data in order to minimize bias, excluding literature from secondary data analyses. Reviewing these studies helps address complex stroke-related questions and solve fundamental experimental issues through novel insights. This manuscript provides an overview of ischemic stroke research using scRNA-seq, offering detailed insights and revealing new features of the disease.

## 2 Changes in cells of brain

### 2.1 Identified cell type from brain and blood

We made a review on the cell type identified from brain tissues and blood. Within the limitation of selective marker, or method itself, from the literature, we got 22 kinds of cell types (1) Vascular smooth muscle cell; 2) Macrophage (including CNS border-associated macrophage); 3) Fibroblast (including perivascular fibroblast-like cell); 4) Monocyte (including monocyte-derived cell); 5) Endothelial cell (including venous endothelial cell, capillary endothelial cell, arterial endothelial cell, vascular endothelial cell); 6) Pericyte (including pericyte, mural cell); 7) Choroid plexus cell (including choroid plexus epithelial cell); 8) Ependymocyte; 9) Microglia (including olfactory ensheathing glial cell); 10) Neutrophil (including dendritic cell neutrophil); 11) Astrocyte; 12) Oligodendrocyte (including oligodendrocyte precursor, border-associated oligodendrocyte precursor cell, oligodendrocyte progenitor cell, oligodendrocyte precursor); 13) Neuron (including neural progenitor cell, neuroblast, neuroendocrine cell, neuronal stem cell, interneuron); 14) Red blood cell; 15) Phagocyte; 16) Eosinophil; 17) Natural killer cell; 18) T cell; 19) B cell; 20) Smooth muscle cell; 21) Epithelial cell; 22) Vascular leptomeningeal cell from brain tissues. Meanwhile 7 kinds of cell types were obtained from blood: 1) B cell; 2) T cell (including CD8^+^ T cell, CD4^+^ T cell, CD4^+^ Treg cell, CD4^+^ memory T cell, macrophage like T cell and populations of CD8^+^ T effector cell, tregs T cell, αβ T cell, γδ T cell); 3) Monocyte (Ly6c monocyte, CD14^+^ monocyte, myeloid dendritic cell, plasmacytoid dendritic cell, classical dendritic cell); 4) Natural killer cell; 5) Neutrophil; 6) Eosinophil. Some cell type have the different names, but has the same cell characteristics, we identified as the same cell type (The specific data and references are shown in Table 1).

**TABLE 1:** The identified cell type from brain tissues or blood after ischemic stroke using scRNA-seq.

The identified cell type from brain tissues after MCAO using scRNA-seq						
Number	Kind	Cell type	Description	Model	Species	Platform
1	17	1) vascular smooth muscle cell; 2) perivascular fibroblast-like cell; 3) CNS border-associated macrophage; 4) monocyte-derived cell; 5) venous endothelial cell; 6) capillary endothelial cell; 7) arterial endothelial cell; 8) pericyte; 9) choroid plexus epithelial cell; 10) ependymocyte; 11) microglia; 12) neutrophil; 13) astrocyte; 14) oligodendrocyte; 15) neural progenitor cell; 16) lymphocyte; 17) red blood cell	The same left cerebral hemispheres from sham-operated and MCAO 24 h mice using 10×Genomics technology (Zheng et al., 2022)	MCAO 24 h	Mice	10×Genomics
2	12	1) microglia; 2) monocyte, 3) macrophage; 4) granulocyte; 5) proliferating cell; 6) natural killer cell; 7) T cell; 8) innate lymphoid cell; 9) B cell; 10) neuron; 11) vascular cell; 12) lymphocyte	The brain tissues from *Prdx1* ^*+/+*^ and *Prdx1* ^*−/−*^ mice after MCAO 24 h using the BD Biosciences (Kim et al., 2022)	MCAO 24 h	Mice	BD Biosciences
3	14	1) ependymocyte; 2) neuroblast; 3) astrocyte; 4) choroid plexus cell; 5) fibroblast; 6) mural cell; 7) T cell; 8) microglia; 9) mononuclear cell; 10) phagocyte; 11) macrophage; 12) endothelial cell; 13) neutrophil; 14) oligodendrocyte	The brain tissues from sham-operated and MCAO 2 h and reperfusion 24 h rat using Singleron GEXSCOPE Single-Cell RNA Library Kit (Zhang et al., 2023a)	MCAO 2 h and reperfusion 24 h	Rat	Singleron GEXSCOPE Single-Cell RNA Library Kit
4	7	1) neuron; 2) ependymocyte; 3) endothelial cell; 4) neuroendocrine cell; 5) oligodendrocyte; 6) astrocyte; 7) oligodendrocyte precursor	The brain tissues from sham-operated and MCAO 1 h and reperfusion 24 h and drug-treated MCAO/R rats using 10×Genomics technology (Cao et al., 2023)	MCAO 1 h and reperfusion 24 h	Rat	10×Genomics
5	7	1) pericyte; 2) neuronal stem cell; 3) oligodendrocyte; 4) neuron; 5) neuronal stem; 6) oligodendrocyte precursor; 7) endothelial cell	The brain tissues from sham-operated and MCAO 1 h neonatal mice using 10×Genomics technology (Frazier et al., 2023)	MCAO 1 h	Mice	10×Genomics
6	9	1) microglia; 2) astrocyte/neural stem cell 3) oligodendrocyte; 4) endothelial cell; 5) macrophage; 6) ependymocyte; 7) T cell; 8) fibroblast; 9) monocyte	The rat cerebral cortex from sham-operated and MCAO 1.5 h and reperfusion 24 h using BD Rhapsody (Zhao et al., 2023)	MCAO 1.5 h and reperfusion 24 h	Rat	BD Rhapsody
7	17	1) microglia; 2) macrophage; 3) endothelial cell; 4) oligodendrocyte; 5) dendritic cell; 6) neutrophil; 7) T cell; 8) astrocyte; 9) choroid plexus epithelial cell; 10) pericyte; 11) B cell; 12) border-associated oligodendrocyte progenitor cell; 13) neuroblast; 14) ependymocyte; 15) fibroblast-like cell; 16) neuron; 17) olfactory ensheathing glial cell	The brain tissues from young adult (12–16 weeks) and aged (20 months) mice under three different conditions: reperfusion 3 days after sham surgery, 3 days and 14 days after MCAO mice using 10×Genomics technology (Jin et al., 2023)	MCAO1 d and reperfusion 3 days and 14 days	Mice	10×Genomics
8	9	1) microglia; 2) oligodendrocyte; 3) endothelial cell; 4) astrocyte; 5) lymphocyte; 6) epithelial cell; 7) vascular leptomeningeal cell; 8) vascular endothelial cells; 9) venous endothelial cell	The brain tissues from young adult (3 months) and aged (20 months) mice underwent sham surgery or permanent stroke using 10×Genomics (Kim et al., 2023)	Permanent stroke	Mice	10×Genomics
9	11	1) astrocyte; 2) B cell; 3) endothelial cell; 4) fibroblast; 5) macrophage; 6) microglia; 7) mural cell; 8) neuron; 9) neutrophil; 10) oligodendrocyte; 11) T cell	The brain tissues from sham-operated and 3 h, 12 h and 3 days after MCAO 1 h mice using 10×Genomics (Liu et al., 2023a)	MCAO 1 h and reperfusion 3 h, 12 h and 3 days	Mice	10×Genomics
10	8	1) microglia; 2) monocyte/macrophage; 3) neutrophil; 4) dendritic cell; 5) natural killer cell; 6) T cell; 7) B cell; 8) type 2 innate lymphoid cell	The brain tissues from young adult (3–4 months) and aged C57BL/6 mice using 10×Genomics (Li et al., 2022)	MCAO 6 h and reperfusion 1 day, 3 days, and 7 days	Mice	10×Genomics
11	13	1) microglia; 2) macrophage; 3) astrocyte; 4) oligodendrocyte; 5) oligodendrocyte progenitor cell; 6) neuron; 7) fibroblast; 8) ependymocyte; 9) B cell; 10) T cell; 11) monocyte; 12) pericyte; 13) endothelial cell	The brain tissues from sham-operated mice and MCAO 1 h and reperfusion 24 h mice using 10×Genomics (Guo et al., 2021)	MCAO 1 h and reperfusion 24 h	Mice	10×Genomics
12	14	1) astrocyte; 2) choroid plexus capillary endothelial cell; 3) endothelial cell; 4) ependymocyte; 5) neuron; 6) macrophage; 7) microglial cell; 8) neutrophil; 9) oligodendrocyte; 10) pericyte; 11) red blood cell; 12) T cell; 13) vascular smooth muscle cell; 14) B cell	The brain tissues from WT mice and *Lrg1* ^*−/−*^ mice under sham-operated and MCAO 1 h and reperfusion 24 h using 10×Genomics (Ruan et al., 2023)	MCAO 1 h and reperfusion 24 h	Mice	10×Genomics
13	8	1) CD4^+^ Treg cell; 2) CD4^+^ memory T cell; 3) natural killer T cell; 4) macrophage like T cell; 5) CD8^+^ T effector cell; 6) B cell; 7) macrophage; 8) neutrophil	The sorted *CD45* ^*high*^ cells from brain tissues of 5 or 14 days after MCAO 1 h mice using 10×Genomics (Shi et al., 2021)	5 or 14 days after MCAO 1 h	Mice	10×Genomics
14	11	1) neuron, 2) oligodendrocyte, 3) astrocyte, 4) CNS border-associated macrophage, 5) monocyte derived cell, 6) neutrophil, 7) lymphoid cell, 8) mural cell, 9) endothelial cell, 10) ependymocyte, 11) perivascular fibroblast like cell	Brains from Sham, MCAO and neutrophil membrane-camouflaged polyprodrug nanomedicine-treated MCAO mice were subjected to MCAO 24 h after drug administration (Zhao et al., 2024)	MCAO 24 h	Mice	NovaSeq 6000
15	10	1) astrocyte, 2) microglia, 3) endothelial cell, 4) oligodendrocyte, 5) oligodendrocyte precursor cell, 6) neuron, 7) natural killer cell, 8) B cell, 9) T cell, 10) granulocyte	Ipsilateral target brain tissues from MCAO, MCAO treated with low-intensity focused ultrasound stimulation, sham or only treated with low-intensity focused ultrasound stimulation were subjected to scRNA-seq analysis (Qi et al., 2024)	MCAO 1.5 h and reperfusion 24 h	Mice	10×Genomics
16	14	1) astrocyte, 2) endothelial cell, 3) fibroblast, 4) microglia, 5) neuron, 6) oligodendrocyte, 7) peripheral myeloid cell, 8) vascular smooth muscle cell, 9) choroid plexus cell, 10) ependymocyte, 11) granulocyte, 12) neuroblast, 13) pericyte, 14) T cell	The study employed 3 month old mice subjected to permanent MCAO and carried out pertinent investigations on the 1, 3, and 7 days following injury (Zucha et al., 2024)	Permanent MCAO and 1, 3, and 7 days following injury	Mice	10×Genomics
17	14	1) fibroblast, 2) ependymocyte, 3) conventional dendritic cell, 4) neuron, 5) CD8^+^T cell, 6) M2 macrophage, 7) choroid plexus cell, 8) neural progenitor cell, 9) smooth muscle cell, 10) endothelial cell, 11) microglia, 12) oligodendrocyte, 13) astrocyte, 14) neutrophil	The intraluminal suture occlusion technique was employed to establish a transient middle cerebral artery occlusion model with 1 h of ischemia followed by 12 h of reperfusion (Wu et al., 2025)	MCAO 1 h and reperfusion 12 h	Mice	Illumina HiSeq X platform
18	8	1) microglia, 2) oligodendrocyte, 3) endothelial cell, 4) pericyte, 5) astrocyte, 6) central nervous system-associated macrophage, 7) T cell, 8) immature neuron	The hippocampal subfields CA1 and CA3-DG of Sprague-Dawley rats were subjected to four-vessel occlusion (4-VO) surgery, a model of transient global cerebral ischemia (Kwak et al., 2025)	Four vessel occlusionsurgery	Rat	10×Genomics
19	9	1) microglia, 2) neuron, 3) natural killer cell, 4) fibroblast, 5) neutrophil, 6) immature neuron, 7) endothelial cell, 8) B cell, 9) neural stem cell	Male 6–8 weeks C57BL/6J mice underwent focal cerebral ischemia induced by intraluminal suture occlusion technique for 1.5 h followed by 24 h reperfusion (Zhang et al., 2025)	MCAO 1.5 h and reperfusion 24 h	Mice	10×Genomics
20	12	1) oligodendrocyte, 2) microglia, 3) endothelial cell, 4) ependymocyte, 5) choroid plexus cell, 6) pericyte, 7) macrophage, 8) astrocyte, 9) neuron, 10) T cell, 11) B cell, 12) fibroblast	Male C57BL/6J mice underwent temporary occlusion of the right common carotid artery using a microvascular clip for 2 h, followed by reperfusion 24 h after clip removal to restore blood circulation (Zhao et al., 2025)	Temporary right vessel occlusion for 2 h with microvascular clip followed by 24 h reperfusion	Mice	10×Genomics
1	8	1) B cell; 2) platelet; 3) CD8^+^ T cell; 4) Treg T cell; 5) proliferation cell; 6) monocyte; 7) natural killer cell; 8) CD4^+^ T cell	The peripheral blood from MCAO 90 min and reperfusion 1 day, 7 days, and 14 days mice using 10×Genomics (Xie et al., 2023)	MCAO 90 min and reperfusion 1 day, 7 days, and 14 days	Mice	10×Genomics
2	10	1) neutrophil; 2) eosinophil; 3) Ly6c monocyte (*Ly6c* ^*lo*^ and *Ly6c* ^*hi*^); 4) dendritic cell; 5) plasmacytoid dendritic cell; 6) B cell; 7) CD4^+^ T cell; 8) natural killer cell; 9) microglia, 10) boundary-associated macrophage	The myeloid cell and neutrophil from blood of mice under MCAO 45 min using 10×Genomics (Gullotta et al., 2023)	MCAO 45 min	Mice	10×Genomics
3	11	1) plasmacytoid dendritic cell; 2) classical dendritic cell; 3) CD14^+^ monocyte; 4) B cell; 5) CD14^+^ monocyte subtype; 6) megakaryocyte; 7) megakaryocyte subtype; 8) CD14^+^ cell; 9) CD16^+^ monocyte; 10) CD14^+^ cell; 11) CD8^+^ T cell	The peripheral blood mononuclear cells from blood of patients who had an acute ischemic stroke within 24 h using NovaSeq 6,000 (Cho et al., 2022)	Acute ischemic stroke 24 h	*Homo sapiens*	NovaSeq 6000

Additionally, some identified cell types in some specific brain tissues are discussed. Cells in hippocampus were identified as 14 kinds of cell types (astrocyte, oligodendrocyte, microglia, monocyte, macrophage, neutrophil, dendritic cell, natural killer cell, T cell, B cell, endothelial cell, neuron, smooth muscle cell, and pericyte). The cell types within cortex were identified as 10 kinds: microglia, astrocyte, oligodendrocyte, endothelial cell, macrophage, ependymocyte, T cell, fibroblast, monocyte, and pericyte (The specific data and references are shown in Table 1).

### 2.2 Cell heterogeneity

During the course of division growth, cells presented molecular characteristic changes in biological or genetic peculiarity, thus generating multiple cellular diversity in the condition or type, which is called cell heterogeneity (Chen et al., 2024; Carter and Zhao, 2021). This apparent cell heterogeneity could be implied for different functions in ischemia disease, like immunity, neuronal enrichment, or neurodevelopment, etc. These classic analysis were achieved via pseudo-time trajectory analysis, including Monocle2, Monocle3, Cytoscape, Slingshot, RNA velocity. Though an attempt is made to stimulate the differentiation of primary cells by Zhang et al. (2019), the whole dynamic process would be difficult to be recorded through basic experiments. scRNA-seq might complete the high resolution analysis to allow computers to simulate cell dynamic evolution trajectory effectively. We have categorized and elucidated heterogeneous cells within cerebral ischemic stroke-related models based on their distinctive cell types related with their associated functions. Additionally, we have compiled and outlined the biomarkers characteristic of these distinct cell subclusters (Supplementary Table 2).

#### 2.2.1 Microglia

**FIGURE 1:**
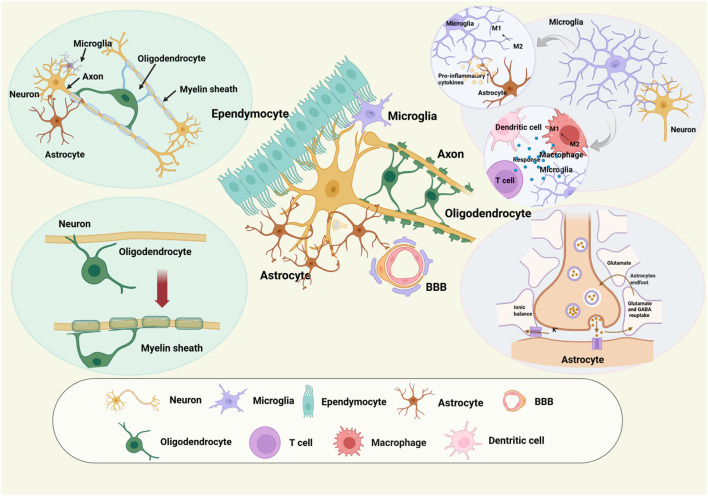
Key functions of microglia, oligodendrocytes, and astrocytes in the aftermath of ischemic stroke, along with their intercellular interactions with other components of the neurovascular unit, including cells of the blood-brain barrier (BBB) and neurons.

Microglia, CNS immune cells, play crucial roles in immune maintenance and response. Within the central nervous system, microglia, macrophages, dendritic cells, T cells, monocytes, and neutrophils, functioning as pivotal elements of the immune system, engage in intricate interactions with oligodendrocytes and astrocytes to coordinate immune responses and safeguard the stability of the brain and spinal cord (Figure 1). Reports on microglia are abundant due to their high distribution, accounting for 0.5%–16.6% of CNS cells. Recent scRNA-seq and lineage tracing have revealed microglial heterogeneity. Discrepancies in microglia subclusters, differentiation pathways, and patterns exist even within the same species under MCAO/R (Xie et al., 2020). These findings have expanded microglial classifications beyond traditional M0, M1, and M2. Spatial localization differentiates ischemic core-associated microglia from penumbra-associated microglia (Liu P.-Y. et al., 2023). Also, ischemic core-associated microglia show higher expression of pro-inflammatory/responsive genes (*Il1a*) and chemokine genes (*Ccl2*) compared to ischemic penumbra-associated microglia (Li et al., 2023).

Li et al. identified 6 microglial subclusters post-stroke, including microglia_0 (homeostatic), microglia_2 (homeostasis-prominent), and microglia_4, 5, 6 (high stroke-gene expression) (Zhang Y. et al., 2023). Post-stroke, *Ch25h* ^*+*^ microglia enhance phagocytosis and neuroprotection, while *Oasl* ^*+*^ microglia accumulate in ischemic brains (Zhang Y. et al., 2023). After stroke, microglia differentiate into 5 subclusters, with 3 subclusters mainly from MCAO patients (Zheng et al., 2022). Increased Hif1a-associated regulon activity was observed across MCAO-dominant clusters (Zheng et al., 2022). Preliminary findings suggest microglia differentiate into 7 clusters post-MCAO/R or *Lrg1* knockout (Ruan et al., 2023). NRN treatment reprogrammed microglia post-MCAO/R, promoting anti-inflammatory polarization (Zhao et al., 2024). Subsequent studies on the effect of NRN on microglial heterogeneity reveal 4 unique subclusters. Post-NRN treatment, microglia_3 and microglia_4 proportions increased significantly versus the MCAO/R group, upregulating neutrophil chemotaxis pathways and downregulating those for neuronal projection development (Zhao et al., 2024). Hao et al. found 5 microglial subclusters in both sham and MCAO groups, with microglia_2 being the most inflammatory and microglia_3 linked to ischemic lesions (Zheng et al., 2022). Zhang et al. reclustered microglia into 14 subclusters, with MCAO samples enriched in inflammation-related subclusters (Guo et al., 2021). In aged brains post-stroke, 6 microglial subclusters exist, with microglia_5 being highly proliferative and microglia_6 mimicking neutrophils (Li et al., 2022). Microglia subclusters show distinct gene expression: microglia_4 for cell clearance/repair (*Spp1*) and chemokines; microglia_5 for proliferation in acute stroke; microglia_3, 4, 7 for disease/neurodegeneration-related genes (*Apoe*) (Han et al., 2024). Another study identified 16 microglia subclusters in mouse brain tissue 3 days post-permanent distal MCAO. Microglial subclustering *P2ry12* ^*+*^ and *Fcrls* ^*+*^ cells revealed reactive microglia heterogeneity. Microglia_4 expressed homeostatic genes, while MCAO-specific microglia_3, 7, and 16 showed high disease-associated gene expression. Microglia_10 and 16 also expressed chemokine/cytokine genes (*Ccl3, Il1b*) (Patir et al., 2024). In the CA1 and CA3-DG of hippocampus, microglia were identified with 7 distinct cell clusters. Under the four-vessel occlusion experiment, the pro-inflammatory cluster 1 predominated in the CA1 region, while the proportion of pro-inflammatory cluster 2 was notably elevated in the CA3-DG region, accompanied by increased phagocytic and neuroprotective scores (Kwak et al., 2025). Interestingly, microglial cell distribution was also examined: Microglial clusters re-clustered into 7 subclusters. In controls, cells mainly gathered in 0 and 5; in MCAO, 0 and 5 decreased, while 1, 2, 3, 4, and 6 increased. Dexmedetomidine suppressed MCAO-induced changes, with microglia_0 decreasing and microglia_1 and microglia_3 increasing (Zhang et al., 2025). Novel microglial subclusters have been identified, such as those with enhanced antioxidant function via peroxiredoxin-1 (Kim et al., 2022) and *Itgb2* ^*+*^ microglia involved in energy metabolism and neuronal interactions (Zeng et al., 2023). Through scRNA-seq studies on microglia in cerebral ischemia, our understanding has greatly improved in two key areas: a nuanced grasp of how microglial heterogeneity shapes inflammatory responses beyond M0, M1 and M2 classifications, and the discovery of its roles in neuroprotection and energy metabolism. Thus, precisely targeting specific microglial subclusters is crucial for future management of brain injury after cerebral ischemia.

#### 2.2.2 Macrophage

CNS-associated macrophages at the blood-brain interface clear debris, present antigens, and participate in inflammation. Hao et al. identified 6 macrophage subclusters at the CNS borders during ischemic stroke, with MCAO-specific subclusters showing distinct features like high MHCII expression and oxidative phosphorylation (Zheng et al., 2022). Post-stroke, monocyte/macrophage clusters increased from 0.5% to 14.9%, with macrophage_1 and macrophage_2 predominant (Li et al., 2022). Han et al. found macrophages are categorized into 4 subtypes: intermediate monocyte/macrophage (*Ly6c2*), high MHCII macrophage (*CD74*), chemokine-enriched macrophage (*Ccl2*), and Arg1-high macrophage (*Arg1*) (Han et al., 2024). In aged mice, infiltration was higher. Peripheral macrophages recruited to ischemic areas polarized into 2 subclusters, with macrophage_2 abundant in MCAO and associated with proinflammatory signals like Tnf-α and IL-6/Jak/Stat3 (Guo et al., 2021). A new *Foxp3+* macrophage subcluster, distinct from M1/M2, enhances efferocytosis post-stroke (Cai et al., 2023). A B-cell-like macrophage phenotype in neuroinflammatory brains exhibits phagocytic and chemotactic functions (Wang et al., 2023). In addition, emerging research has elucidated the correlation between clinical patient datasets and macrophage phenotypes. Specifically, in patients with acute cerebral ischemia, heightened peripheral levels of Tie2-expressing macrophages are strongly associated with improved clinical prognoses, implying that targeting these Tie2-high macrophages in the systemic circulation might constitute a pivotal mechanism mediating the brain’s intrinsic neuroprotective response (Sheng et al., 2021). Utilizing scRNA-seq analysis, it has been revealed that the principal functions of macrophages encompass phagocytosis and chemotaxis. CNS-associated macrophages at the blood-brain barrier show diverse subclusters traits in ischemic stroke, enabling precise, targeted interventions via their distinct markers.

#### 2.2.3 Oligodendrocyte

Oligodendrocytes constitute a vital element of the brain’s white matter. Brain ischemia can induce demyelination due to oligodendrocyte injury, a process that readily provokes neuroaxonal degeneration, thereby ultimately giving rise to neurobehavioral changes and sensorimotor impairments (Figure 1). MCAO induces a reactive oligodendrocyte subcluster aiding white matter repair post-ischemic stroke in early ischemic striatum (Frazier et al., 2023). These *Olig2* ^*+*^ *Edu* ^*+*^ oligodendrocytes proliferate 3–7 days post-MCAO, but don’t fully mature by day 28, reducing myelinated axons, which might be a therapeutic target for enhancing repair (Frazier et al., 2023). The MCAO group has higher enrichment of all 9 oligodendrocyte subclusters, especially subcluster 9, in inflammatory, interferon-gamma, complement system, and IL-6/Jak/Stat3, TNF-α, NF-κB, IL-2-Stat5 pathways (Guo et al., 2021). Metabolic scoring shows metabolite alterations in oligodendrocytes early post-stroke (Guo et al., 2021). Researches revealed that differentially expressed genes downregulated in the oligodendrocyte subclusters from the MCAO contralateral side compared to the sham group encompass neurexins and neuregulins (*Nrxn1, Nrxn3, Nrg1, Nrg3*), along with genes encoding neurotransmitter receptors, ion channels, and ion channel-interacting proteins (*Kcnip4, Grm5, Kcnq5*) (Bormann et al., 2024). Within the hippocampus under four-vessel occlusion experiment, oligodendrocytes were partitioned into 4 distinct cell subclusters. Under sham-operated conditions, solely the oligodendrocyte_1, 2, 3 cell clusters were detected. Conversely, under four-vessel occlusion condition, there was an augmentation in the proportion of the oligodendrocyte_1, and exclusively the oligodendrocyte_4 was identified, which displayed gene expression patterns linked to apoptotic pathways and leukocyte activation, specifically expressing *Sult1a1* in immature oligodendrocytes (Kwak et al., 2025). Oligodendrocytes are crucial for brain white matter integrity. Brain ischemia damages oligodendrocytes, causing demyelination, neuroaxonal degeneration, neurobehavioral, and sensorimotor disorders. scRNA-seq reveals metabolic changes in oligodendrocytes during ischemia, with the *Olig2* ^*+*^ *Edu* ^*+*^ oligodendrocyte subclusters showing promise for targeted therapies.

#### 2.2.4 Astrocyte

After cerebral ischemic stroke, activated astrocytes play a pivotal role in influencing neuronal survival and facilitating repair. The modulation of calcium and potassium ion channels emerges as a crucial target in this context. Furthermore, astrocytes might undergo morphological alterations, including the rounding of cell bodies and marked shortening of processes, accompanied by an increase in glutamate and aspartate concentrations within the synaptic cleft. These astrocytes mediate neurovascular interactions by engaging in intercellular communication through gap junctions (Figure 1). Seven distinct astrocyte subclusters were discerned within the brain following the induction of ischemic stroke (Guo et al., 2021). Unlike microglia/macrophages, astrocyte subcluster changes between MCAO and sham groups were less pronounced. However, astrocyte_3 showed greater diversity and metabolic enrichment in O-glycan biosynthesis and thiamine metabolism. Early stroke astrocyte responses focus on intracellular regions, metabolic processes, and MAPK signaling (Guo et al., 2021). Another study classified astrocytes into three subclusters. Astrocyte_B had a higher proportion than astrocyte_C, while MCAO samples showed the opposite. Astrocytes transitioned from dormancy (astrocyte_A) into 2 functional states: astrocyte_B (expressing *Itm2a, Dbp*) and astrocyte_C (expressing *Spp1, Ccl4*). In addition, *Aldoc* expression in astrocytes across injured and uninjured conditions supports its role as a reliable pan-astrocyte marker (Scott et al., 2024). After cerebral ischemia-reperfusion, astrocytes differentiate into 4 subclusters. *Nes* and *Ascl1*, marker genes of A2-type astrocyte, show notably high expression in A2-type astrocytes post-MCAO, minimal to none in the A1-type astrocyte group, implying a neurotrophic function, and both genes are unexpressed in the sham-operation group (Wu et al., 2025). Within the CA1 and CA3-DG of hippocampus under four-vessel occlusion condition, astrocytes were delineated into 2 cell clusters. Astrocyte subcluster 1 manifested functions pertinent to angiogenesis and vascular development, whereas astrocyte subcluster 2 demonstrated functions associated with synaptic assembly and organization (Kwak et al., 2025). Astrocytes, with abundant processes in nerve cell interspaces, support, separate, and help form the blood-brain barrier. After cerebral ischemic stroke, their activation is crucial for neuronal survival and repair, accompanied by morphological changes and neurovascular interaction mediation. Currently, scRNA-seq analysis on astrocytes is limited, mainly preliminary in exploring differentiation and subcluster functions.

#### 2.2.5 Dendritic cell

Dendritic cells, which are specialized in presenting antigens, have received limited attention in stroke research. However, Yan et al. demonstrated a significant influx of dendritic cells into the brain following ischemic stroke. Remarkably, the dendritic cell subcluster showed elevated expression of myosin heavy chain class II-associated genes, including *H2-Aa, H2-Ab1,* and *CD74*, implying a possible role in antigen presentation related to the pathophysiology of ischemic stroke (Li et al., 2022). Dendritic cells remain understudied in stroke research, yet current findings link them to antigen presentation within the pathophysiological mechanisms of ischemic stroke.

#### 2.2.6 T cell

Among patients with acute ischemic stroke, specific T cell subclusters could fuel the progression of inflammation and exacerbate ischemic damage. T cell subclusters have complex roles, with some fueling inflammation and others exhibiting neuroprotective effects. Notably, scRNA-seq has unveiled the immunomodulatory potential of regulatory T cells (Shi et al., 2021). Treg-derived osteopontin aids microglia-driven white matter repair. Mouse studies show Treg cells migrate to the brain 1–5 weeks post-stroke, and their elimination hinders oligodendrogenesis, repair, and recovery. Notably, it is the depletion of microglia, rather than a general reduction in T cells, that diminishes the beneficial effects of Treg cells on white matter regeneration (Shi et al., 2021). scRNA-seq reveals T cell distribution and crosstalk in post-ischemic brain tissue. Acute ischemic stroke patients show distinct T cell subclusters with dual roles: some drive inflammation/ischemic damage, others confer neuroprotection—thus necessitating precise customization of targeted therapies.

#### 2.2.7 Monocyte

Following an ischemic stroke, monocytes migrate from the bloodstream to damaged tissues; this infiltration is a critical immune response that drives wound healing and tissue repair. scRNA-seq and pseudo-time analysis of stroke patient blood revealed 18 monocyte subclusters and 2 branching points (Xie et al., 2023). Monocyte cluster 10 spans the pseudotime trajectory, suggesting a pivotal role in differentiation. *Apoe, Csf1r,* and *Ctss* genes are crucial for monocyte activation after cerebral ischemia-reperfusion (Xie et al., 2023). Despite limited monocyte research, scRNA-seq has identified key target genes for activating critical inflammatory monocyte subsets.

#### 2.2.8 Neutrophil

**FIGURE 2:**
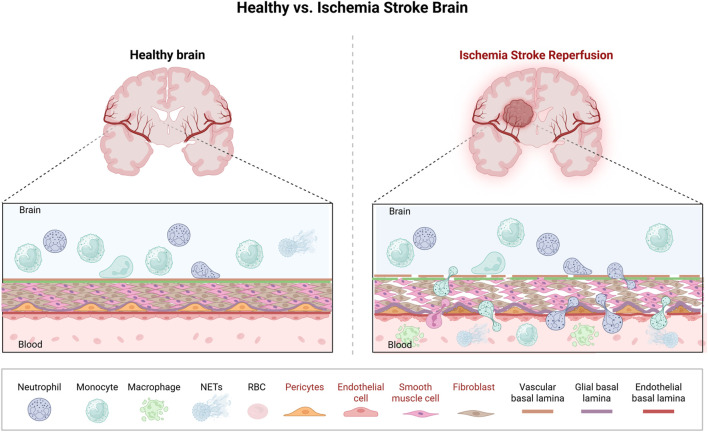
A schematic diagram presents the changes in neutrophils associated with BBB damage. Following ischemic stroke, endothelial cells, vascular smooth muscle cells, pericytes, and fibroblast-like cells demonstrate impaired inductive and contractile functions, resulting in cell loss and vascular stenosis. This pathology restricts cerebral blood flow and disrupts the integrity of the blood-brain barrier. Concurrently, neutrophils engage with endothelial cells through a sequential process involving rolling, adhesion, and crawling, culminating in transmigration across the BBB and recruitment to the ischemic brain region.

After ischemic stroke, neutrophils quickly reach the brain, peaking at 24 h, disrupting the BBB, causing edema, and generating oxidative stress (Figure 2). Four neutrophil clusters were identified in the brain, with neutrophil_0, the most mature, highly expressing Cxcl1 (Zheng et al., 2022). scRNA-seq revealed 6 neutrophil clusters in the blood (Gullotta et al., 2023), with age-related changes leading to atypical neutrophils exacerbating stroke. Aged mice had higher Cxcl3 production by *CD62l* ^*low*^ neutrophils, linked to poorer reperfusion and outcome. Elevated plasma levels of neutrophil growth factors and proinflammatory mediators were also seen in aged mice. Yang et al. distinguished three neutrophil subsets in the brain, with neutrophil_1, composed of infiltrated neutrophils, increasing from 0.3% to 18.7% post-stroke. Neutrophil_1 cells showed high activation, with neutrophil_2 categorized as immature (Li et al., 2022). scRNA-seq reveals neutrophil heterogeneity post-cerebral ischemia, including maturity and chemokine variations linked to cerebral edema and oxidative stress, promising future identification of specific subclusters and targeted therapies.

#### 2.2.9 Neuron

Neurons that undergo changes due to conditions as ischemic stroke or cerebral hemorrhage. Fourteen glutamatergic neurons were collected from both sham-operated and infarct brains 4 days post-ischemic stroke (Nakamura et al., 2023). Post-ischemic stroke, glutamatergic neurons change and show potential as systemic targets, with *Padi4* ^*flox/flox*^ mice in peri-infarct neurons promoting recovery via histone citrullination, confirmed by immunohistochemical analysis (Nakamura et al., 2023). Six neuron subclusters were identified (Guo et al., 2021), with neuron subcluster 4 enriched in MCAO groups focusing on fatty acid metabolism, glycolysis, gluconeogenesis, and amino acid metabolism, including gamma-aminobutyric acid and dopamine neurons in specific subclusters (Guo et al., 2021). Mildner et al. found a canonical stress response emerged in the ambiguous GABAergic neuronal cluster, marked by upregulation of heat shock proteins (*Dnaja1, Hsp90aa1, Hspa8, Hsph1*). *Adarb2* includes glutamatergic interneurons, as well as *Satb2* includes in glutamatergic neurons (Bormann et al., 2024). scRNA-seq-based identification of neuronal subclusters post-cerebral ischemia encompasses GABA-predominant inhibitory neurons, glutamate-predominant excitatory neurons, and interneurons, all of which could function as potential target cells for reversing neural injury after cerebral ischemia.

#### 2.2.10 Endothelial cell

Endothelial cells, vascular mural cells (encompassing pericytes or rouget cells), perivascular fibroblast-like cells, and vascular smooth muscle cells are interconnected components within the vascular wall that collectively maintain vascular health and function. Endothelial cells line the lumen of blood vessels and serve as a critical interface between the blood and the surrounding tissues. Perivascular fibroblast-like cells reside in the adventitial layer of blood vessels and are involved in extracellular matrix production. These cells communicate with endothelial cells and mural cells to coordinate vascular repair and maintenance. Vascular smooth muscle cells, located in the media of blood vessels, regulate vessel diameter and blood flow through contraction and relaxation, thereby controlling blood pressure and tissue perfusion (Figure 2). Six endothelial cell subclusters were identified, with BBB-associated clusters showing increased dysfunction-related genes in MCAO (Zheng et al., 2022). Capillary and artery clusters expressed interferon-I genes, but proportions decreased in MCAO (Zheng et al., 2022). Endothelial cell subcluster 3, enriched in BBB features, triggers apoptosis in cerebral endothelial cells during stroke. Endothelial cell subcluster 5 is linked to hypoxia and oxidative phosphorylation (Zheng et al., 2022). Post-stroke, a transient angiogenic cluster emerges in younger brains but diminishes with age, accompanied by strong interferon responses and reduced proangiogenic capabilities (Jin et al., 2023). Aging might enhance apoptosis and disrupt endothelial circadian rhythms (Jin et al., 2023). While MCAO and sham-operated groups had similar subcluster proportions, subcluster 3 showed elevated epithelial-mesenchymal transition and myogenesis capacity, involved in vascular remodeling and extracellular communication post-injury (Guo et al., 2021). Leucine rich alpha-2-glycoprotein 1 marks early post-stroke reactive endothelium. Endothelial cells are divided into 9 subclusters associated with venous capillaries, large veins, arterial capillaries, and arteries, exhibiting metabolic and proliferation characteristics as well as fenestrated brain traits at 2 and 14 days post-stroke (Garcia-Bonilla et al., 2024). Notably, certain studies have specifically investigated the spatial distribution patterns and quantitative characteristics of endothelial cells. After cerebral ischemia-reperfusion, endothelial cells re-clustered into 7 subclusters, with clusters 0–3 making up >93%. Compared to sham group, the MCAO group with dexmedetomidine had fewer endothelial cells. Endothelial cluster 3, linked to oxidative phosphorylation, suggests dexmedetomidine might improve mitochondrial function and correct disturbances (Zhang et al., 2025).

Endothelial cells are not just a barrier but an organ (Bloom et al., 2023). Research on their heterogeneity yields precise cell subclusters for disease treatment. Additionally, researchers have conducted blood-brain barrier intervention studies. Lemin Zheng’s team used scRNA-seq on mouse cerebral cortex tissues, classifying BBB-related cell subclusters. They found connexin 43 was concentrated in these subclusters, with decreased expression in elderly mice, and proposed interventions like olaparib to inhibit Parp1 or nicotinamide mononucleotide supplementation (Zhan et al., 2023). Another research team uncovered that Wnt7a and Wnt7b play crucial roles in regulating the development and integrity of the blood-brain barrier. However, the broad expression profile of Wnt proteins has raised safety concerns regarding their therapeutic application. They engineered Wnt7a for specific binding with Gpr124 and Reck agonists (Martin et al., 2022). Identifying precise endothelial or BBB cell subclusters and protein targets will scientifically support biological drug development.

#### 2.2.11 Vascular mural cells or pericyte or rouget cell

During ischemic conditions, 3 pericyte subsets were identified (Zheng et al., 2022). Vascular mural cell subcluster maintains normal BBB functions through ion transport processes. Vascular mural cell subcluster 1 shows heightened immune gene expression post-stroke, including oncostatin-M-triggered BBB disruption, Hif1 signaling, and cytokine-mediated pathways. Vascular mural cell subcluster 3, expressing high Acta2, governs cerebral blood flow through muscle contraction and the syndecans 1 pathway (Zheng et al., 2022). Pericytes play a pivotal role within the brain’s neurovascular unit, where they are strategically positioned within the capillary endothelial basement membranes, facilitating communication through direct cell-cell contact and paracrine signaling mechanisms. Pericytes respond to stroke early, ahead of endothelial cells and BBB disruption (Roth et al., 2025). Our group previously noted occasional difficulty in distinguishing pericytes from endothelial cells, with limited scRNA-seq reports on pericytes currently (Roth et al., 2025).

#### 2.2.12 Perivascular fibroblast-like cell

In the brain’s perivascular space, a newly identified subset of perivascular fibroblast-like cells has undefined functions (Zheng et al., 2022). Research has identified 3 perivascular fibroblast-like cell subclusters: cluster 0 expresses genes related to membrane transporters and collagen organization; cluster 1 is enriched in extracellular matrix and interferon-beta response genes; cluster 2 highly expresses genes encoding pumps, carboxylic acid transport, and cellular pH regulation (Zheng et al., 2022). Recent studies have demonstrated that the interplay between fibroblasts and immune cells constitutes a pivotal regulatory phase in brain injury repair, where a dysfunctional equilibrium in their states may result in the failure of repair mechanisms or the onset of chronic inflammation (Ewing-Crystal et al., 2025). Nevertheless, the existing literature on fibroblast subclusters and their functions, as delineated by scRNA-seq, remains notably sparse.

#### 2.2.13 Vascular smooth muscle cell

Vascular smooth muscle cells, serving as the predominant cell type in the tunica media of the vascular wall, not only play a pivotal role in maintaining vessel structure and function but also possess metabolic and endocrine functional properties. In normal states, they contract/relax to regulate vascular caliber, controlling blood flow/pressure, and secrete extracellular matrix components for vessel formation/repair. In ischemic conditions, 6 smooth muscle cell subsets emerged, including arteriole, arterial, and venous clusters (Zheng et al., 2022). Subcluster 4 of activated cells was enriched in neutrophil-mediated immune responses and exocytosis regulation, while vascular smooth muscle cell subcluster 5, linked to type I interferon, had a reduced proportion in the MCAO group (Zheng et al., 2022). The exploration of smooth muscle cells through scRNA-seq remains in the nascent phases of scientific inquiry.

#### 2.2.14 Other cells

Other studies suggest heterogeneity in ependymal and choroid plexus capillary endothelial cells (Li et al., 2022). Both cell types share high homology with known genes (Yao et al., 2023). Research categorized ependymal cells into 6 subclusters and endothelial cells into 2 subsets. Ependymal cells are highly expressed in MCAO groups, correlating with cell secretion, ciliary movement, immune response genes, and chemotaxis (Li et al., 2022). Stroke-associated myeloid cells exhibit a mixed macrophage-microglial phenotype, sharing markers with both (*Apoe, Lyz2, Tmem119*) and uniquely expressing high levels of *Spp1, Fabp5, Gpnmb,* et al. At 72 h post-ischemia, stroke-associated myeloid cells slightly upregulate macrophage-related genes, indicating specialized, activated myeloid cells with lysosomal and phagocytic activity resembling embryonic microglia in lipid debris clearance (Beuker et al., 2022). Stroke-associated myeloid cells show a mixed macrophage-microglial phenotype, and uniquely expressing high *Spp1, Fabp5*, etc. At 72 h post-ischemia, they upregulate macrophage-related genes, suggesting specialized, activated cells with lysosomal/phagocytic activity akin to embryonic microglia in lipid clearance (Beuker et al., 2022). Eight fibroblast subclusters were identified, with the majority of fibroblasts enriched in subclusters 0–3. Fibroblast subcluster 1 presented antigens, while subcluster 3 responded to oxidative stress and regulated immunity. This confirmed MCAO upregulated fibroblast genes for antigen presentation, immune responses, and oxidative stress. Dexmedetomidine pretreatment reduced oxidative stress and immune responses, protecting the brain from BBB damage after MCAO/R (Zhang et al., 2025).

## 3 Stroke-induced immune response

**FIGURE 3:**
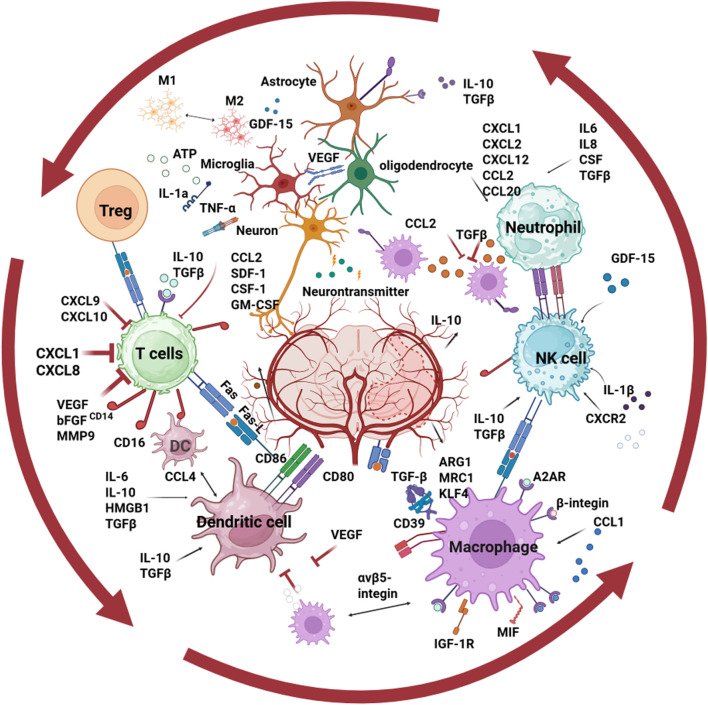
The interactions among immune cells in the brain following ischemic stroke.

Ischemic stroke alters microglia, initiating an immune response involving innate and peripheral immunity. Leukocytes, including neutrophils, monocytes, dendritic cells, and lymphocytes, migrate to the ischemic brain. Understanding distinct immune subsets is a promising therapeutic target. Our attention is directed towards the regulatory influence 1 cell has on another (Figure 3), as indicated by existing literature, as described in literature, not individual ligand-receptor bindings.

Intercellular communication plays a vital role in the immune response elicited by ischemic stroke. In the penumbra post-stroke, activated microglia aid macrophage extravasation and disrupt the BBB by engulfing endothelial cells (Jolivel et al., 2015). Recent research has unveiled new microglia-lymphocyte interactions in ischemic stroke, particularly the Spp1-Ptger4 pathway involved with macrophage-exhausted CD8^+^ T cells (Zheng et al., 2022). Microglia release IL-1α and Tnf-α, inducing A1 astrocyte differentiation (Liddelow and Barres, 2017; Liddelow et al., 2017). Post-stroke, M2 microglia-derived vesicles inhibit astrocyte proliferation and reduce glial scars, enhancing recovery (Li et al., 2021). M2 microglia, derived from M1 post-stroke, facilitate oligodendrocyte progenitor differentiation, crucial for remyelination (Bain et al., 2013; Miron et al., 2013). Astrocytes prevent microglia overactivation via Tgf-β signaling and facilitate monocyte/macrophage infiltration (Norden et al., 2014; Tei et al., 2013). Treg cells modulate other immune cells, and microglia depletion reduces their benefits on white matter regeneration (Zheng et al., 2019). After cerebral ischemia, monocyte-derived macrophages activate progressively and interact with anti-inflammatory, tissue-repair-promoting microglia. Through mechanisms involving Sox2 and Akt/Creb signaling pathway, they modulate neuroinflammatory responses, aiding neural repair (Liu et al., 2024). Neutrophil extracellular traps released lipocalin 2 could induce astrogliosis, which represents the core pathogenic mechanism underlying emotional disorders following cerebral ischemia. This finding elucidates a unique peripheral-central neuroimmune interaction pattern after blood-brain barrier damage (Liu et al., 2025). Neurons and glial cells maintain CNS balance through complex signaling (Gupta et al., 2018). Oligodendrocytes form myelin sheaths around axons (McTigue and Tripathi, 2008). Post-stroke, microglia and macrophages promote angiogenesis and oligodendrogenesis, but oligodendrocyte maturation is disrupted, hindering remyelination (Jin et al., 2023; Marin and Carmichael, 2019). Microglia influence oligodendrocyte progenitor cells, with Vegf stimulating proliferation and inflammatory factors harming them (Ma et al., 2017; Deng et al., 2008). Fibroblasts modulate immune responses in chronic infections, inflammation, and cancer (Davidson et al., 2021). Homma et al. found reduced fibroblast-like cell interactions in MCAO groups compared to sham (Zheng et al., 2022).

Neurons and glial cells maintain CNS balance through complex signaling pathways. In the four-vessel occlusion model, significant interactions were observed between T cells and other cell types, such as oligodendrocytes, endothelial cells, pericytes, and immunoneurons, particularly within the CA3-DG region (Kwak et al., 2025). Neuronal death triggers microglial activation post-stroke, while healthy neurons inhibit it via Cx3cl1/Cx3cr1 signaling (Kriz and Lalancette-Hébert, 2009; Schwartz, 2003; Lauro et al., 2019). Activated microglia accelerate neuronal damage via M1 phenotype-induced mitochondrial fission and cytokine storms, but also stimulate neuroprotective effects (Liu et al., 2022). Similarly, neurons can impact microglial function, as ischemic neuronal damage stimulates microglial cells to exert neuroprotective effects (Ma et al., 2017). Astrocytes aid neuronal regeneration post-stroke but form glial scars hindering neuronal reconnection and disrupting the BBB (Revuelta et al., 2019; Colombo and Farina, 2016). Oligodendrocytes create myelin sheaths, but their maturation is disrupted post-stroke (Revuelta et al., 2019; Colombo and Farina, 2016). Damage signals activate neurons and glia, causing astrocytes to release molecules that disrupt the BBB and worsen injury, but also protective neurotrophic factors (Amantea et al., 2015).

Age alters intercellular communication post-stroke in mice. In young mice, *Vegfa, Gdf15, Igf1, Mif* mediate microglia/macrophage-endothelial interactions on days 3 and 14, but this changes in aged mice. Vegfa exerts its proangiogenic effects in the young brain by acting through microglia/macrophages, thereby influencing endothelial cells (Greenberg and Jin, 2013). Mif-Ackr3 interaction, crucial for angiogenesis (Amin et al., 2003), is absent in aged brains due to low Ackr3. Aging disrupts repair-associated interactions between microglia/macrophages and oligodendrocytes (Jin et al., 2023; Amin et al., 2003). Myeloid cells, especially microglia expressing immediate-early genes, facilitate signal secretion (Liu et al., 2024). Monocyte-derived macrophages interact with microglia via selectins (Liu et al., 2024).

Although research on the mutual regulatory dynamics between immune cells in brain tissue and blood after cerebral ischemia remains a hotspot, the clinical application of specific cell subclusters or targets identified via scRNA-seq is still limited. During ischemic stroke, T cells and activated microglia show enhanced synergistic interactions. Th1, Th17 cells, and M1 microglia secrete pro-inflammatory cytokines like interferon-γ, tumor necrosis factor-α, and interleukin-1β, promoting neuroinflammation, exacerbating brain injury, and affecting patient prognosis. Literature cited in the article indicates that clinical trials have used the immunosuppressant fingolimod or infused excessive regulatory T (Treg) cells to modulate T cell-microglia interactions, aiming to facilitate anti-inflammatory signaling, foster neural tissue regeneration, and reduce ischemic stroke damage. However, such research is scarce, and clinical experience is lacking (Zheng Y. et al., 2025). Myeloid-derived monocytes/macrophages with high Tie2 expression can stimulate endogenous angiogenesis in the mouse brain after ischemic injury, and their peripheral blood abundance correlates with clinical outcomes (Sheng et al., 2021). Furthermore, research has investigated the mechanisms of specific clinical drug interventions for cerebral ischemia, highlighting their potential effects. Pretreatment with dexmedetomidine markedly reduces cerebral injury in MCAO mice, regulates hexokinase 2-expressing microglia’s phagocytosis, and alleviates immune cell decline, including neutrophils, B cells, and antigen-presenting fibroblasts (Zhang et al., 2025).

Additionally, for other neurological disorders like central nervous system lymphoma, a novel drug delivery platform using the gut-brain axis non-invasively bypasses physiological barriers for targeted macrophage delivery to the brain in response to *Cx3cr1* (Gong et al., 2025). Newly discovered cell subsets are closely linked to inflammation and phagocytosis, a crucial area for ischemic stroke treatment. Companies like General Electric have isolated specific cell subsets to develop drugs, including antibodies against specific antigens. Examples include Natalizumab (2004) for MS and Crohn’s, Alemtuzumab (2014) for recurrent multiple sclerosis, Lecanemab (2023) for mild Alzheimer’s disease, Inebilizumab (2016) for neuromyelitis spectrum disorder, Satralizumab (2020) as an IL-6 receptor antagonist for neuromyelitis spectrum disease, and Efgartigimod Vyvgart (2021) for autoantibody IgG.

In the realm of basic research, while scRNA-seq excels at elucidating cellular immune functions, it might still lack the precision to comprehensively delineate the dynamic fluctuations in immune responses. Nevertheless, there persists a keen interest in deciphering the evolutionary patterns of immune communication between the brain and the circulatory system. Concurrently, in clinical research, despite a plethora of scRNA-seq investigations into immune cell interactions after cerebral ischemia, the translation of these findings into clinical practice remains constrained, underscoring the need for further exploration in biological drug delivery.

## 4 Stroke-induced other cell-cell communication

**FIGURE 4:**
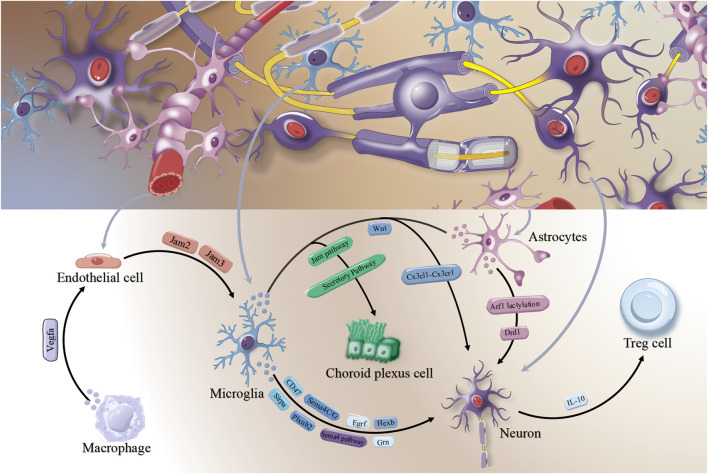
The interactions among other cell-cell communication besides immune cells in the brain following ischemic stroke.

Beyond well-known immune cell interactions, stroke also triggers complex non-immune cell interactions in the brain. Literature review shows that after cerebral ischemia, endothelial and neuronal cells act as key hubs for these interactions. They dynamically interact with immune cells like microglia, astrocytes, and T cells through multiple signaling pathways. These interactions significantly and undeniably impact stroke pathology, neural repair, and functional recovery. Firstly, among non-immune cells, endothelial cells emerge as the primary hubs of interaction, engaging in extensive and intricate crosstalk with neurons, macrophages, and microglia. In the post-stroke brains of young mice, the vascular endothelial growth factor secreted by macrophages exerts effects on endothelial cells. Conversely, in aged brains, 14 days following a stroke, the expression of Vegfa in macrophages diminishes, leading to the suppression of interactions between these 2 cell types. Simultaneously, endothelial cells in the aged post-stroke brain nearly entirely lose Ackr3 gene expression, thereby becoming incapable of receiving macrophage migration inhibitory factor signals released by microglia (Jin et al., 2023). The interaction between Jam2 and Jam3 promotes the adhesion of microglia to endothelial cells and orchestrates their communication with pericytes (Ma et al., 2023). In permanent MCAO model mice, during glial scar formation at severely impaired lesion peripheries, myeloid and endothelial cells dominated lesions, showing strong correlation with microglial activation markers (Figure 4). A coordinated mechanism involving microglia, myeloid cells, and endothelial cells was thus established (Zucha et al., 2024).

The second hub cell type in pathophysiological processes is neurons. Neuronal injury subsequent to cerebral ischemia plays a pivotal role in the progression of ischemic brain damage, with its underlying mechanisms encompassing a spectrum of interconnected processes, including energy metabolism failure, excitotoxicity mediated by amino acid neurotransmitters, intracellular calcium overload, oxidative stress, and the activation of apoptotic signaling cascades. Beyond these well-established molecular mechanisms, recent studies reveal that dynamic immune cell changes and their complex interactions with neurons are novel mechanisms influencing neuronal injury. Microglia exhibit abundant interactions associated with neurotrophic support, involving signal regulatory protein alpha, plexin B2, and progranulin respectively communicate with neurons through CD47, Sema4C/G, and epidermal growth factor receptor (Ma et al., 2023). In the peri-infarct region, microglia associated with axon tracts are closely related to neuronal regeneration. They interact with neural lineage cells via the sema pathway, which promotes neurogenesis (Liu et al., 2024). Also, a novel interaction mechanism between microglia and neurons is revealed. Under physiological homeostasis, microglia deliver β-hexosaminidase to neurons, degrading ganglioside GM2 in them (Figure 4). This preserves neuronal membrane integrity and function, mitigating neurodegenerative pathologies (Frosch et al., 2025). Neuron-targeted ROS-responsive liposomes delivered puerarin, reducing neuronal mortality and markedly shrinking cerebral infarction volume via microglia modulation (Chen et al., 2025).

Another documented modulatory effects was exerted by astrocytes on neuronal function and viability. The conditional ablation of dopamine D1 receptors in astrocytes potentiates glutamatergic synaptic transmission and long-term potentiation within pyramidal neurons of the medial prefrontal cortex (Yin et al., 2025). Following cerebral ischemia, low-density lipoprotein-related receptor protein 1 modulates the lactylation modification of ADP-ribosylation factor 1, consequently governing the intercellular mitochondrial transfer between astrocytes and neurons and mitigating neuronal injury post-cerebral ischemia (Zhou et al., 2024). In brain organoid models, protein- and nutrient-rich astrocyte conditioned medium accelerates neuronal differentiation, thickens the neuronal layer, and boosts deep-layer cortical neuron production. Astrocyte secreted signaling molecules also promoted lipid droplet accumulation in neural cultures, protecting neural differentiation from cellular stressors (Zheng H. et al., 2025). In addition, there are also reports on the complex interactions among microglia, astrocytes, and neurons. The decline in astrocyte-synapse contact preceding microglia-mediated synapse engulfment relies on the secretion of Wnt proteins by microglia, which occurs downstream of the neuronal-microglial chemokine ligand-receptor (Cx3cl1-Cx3cr1) signaling cascade, contributing to synaptic remodeling (Faust et al., 2025) (Figure 4). Research also revealed that regulatory T cells residing within ischemic brain tissue attenuate the excessive and prolonged hyperexcitability of peri-infarct neurons through the IL-10 signaling pathway (Schmidt-Pogoda et al., 2025).

Furthermore, post-cerebral ischemia, intricate interactions occur between immune cells and non-immune cells within the brain microenvironment. In comparison to the sham-operated cohort, the MCAO group displayed a significantly elevated degree of intercellular interactions, which were mediated by the junctional adhesion molecule pathway, among astrocyte clusters 1, 2, and 4, microglia subcluster 1, and choroid plexus cells (Wu et al., 2025). In stark contrast, within the sham-operated group, astrocyte cluster 2 showed few interactions with those two specific cell types. Moreover, within the MCAO group, reciprocal interactions via the trophic factor secretion pathway were markedly intensified between microglia subclusters 1 and 2, choroid plexus cells, and astrocyte subcluster 1 (Wu et al., 2025). After transient MCAO, profibrotic myofibroblasts infiltrate the site of injury brain, when deprived of the chemokine Cxcl12, could disrupt the late-stage lymphocyte-fibroblast niche. This leads to brain-specific late-stage innate inflammation, causing lymphocyte dispersion, exacerbating subacute brain injury and secondary neuroinflammation, and ultimately increasing mortality (Ewing-Crystal et al., 2025).

## 5 Age related issues in ischemic stroke

Theory explaining age-related differences in ischemic stroke in mice is a research hotspot. While cell types are similar, functional enrichment in distinct subgroups differs, with emphasis on senile endothelial cells (Kiss et al., 2020). Aged brains show a 10% higher senescence gene enrichment score in endothelial cells and microglia, with a consistent neuron proportion (Kiss et al., 2020). A unique neutrophil-like microglial subtype (cluster 6) may be stroke-specific (Kiss et al., 2020). Elderly stroke brains show increased microglia and lymphocyte proportions, and decreased oligodendrocyte proportions (Kim et al., 2023). Myeloid cell infiltration is higher in aged mice (Li et al., 2022). In addition, in experimental stroke, neutrophil blockages in ischemic cerebral microcirculation increased in older mice compared to young mice, *CD62l* ^*low*^ neutrophils as predominant neutrophil, resulting in complicating blood flow and worse prognosis (Gullotta et al., 2023). Microglia and macrophages induce angiogenesis and oligodendrogenesis post-stroke, but this is disabled in aged brains (Zhao et al., 2023; Jin et al., 2023).

Regarding scRNA-seq based cerebral ischemia interventions, our team proposes two strategies for aging-related genes. First, drugs or gene therapies targeting aging genes in endothelial cells and microglia (with high enrichment in aging brains) could mitigate post-stroke brain damage. Second, as elderly stroke patients show increased microglia/lymphocytes and decreased oligodendrocytes, methods to restore cell population balance are crucial. Although aging microglia and endothelial cells are reported, specific interventions are lacking. An alternative intervention strategy centers on intercellular communication. Through the repair of damaged myelin sheaths in the context of aging and diverse neurodegenerative disorders, it becomes feasible to ameliorate the clinical manifestations associated with these conditions. Studies indicate that microglia could facilitate myelin regeneration by curbing the recruitment of peripheral monocytes (Kent and Miron, 2024). Furthermore, authoritative studies have demonstrated that microglial activation is a common observation across a range of neurodegenerative diseases, implying that targeting microglial activity might represent an innovative therapeutic approach (Tripp et al., 1986).

## 6 Advantage and disadvantage

### 6.1 Advantage

scRNA-seq in brain science highlights our limited knowledge of cell types and reveals targets influencing multiple cell types. It identifies accurate cell subclusters in ischemic stroke and saves time and cost on lab tests, facilitating for search-like queries in targets. Cost reductions and technological advances make scRNA-seq routine for target identification, with improved cellular resolution crucial for mapping the human cell cycle. scRNA-seq technology has developed from single-tube and multi-well plates to droplet microfluidics and combined tags, leading to a rapid increase in the throughput of sequencing cells and consequently improved cellular resolution, which is the key to mapping the human cell cycle atlas. Of all the potential methods available, scRNA-seq is currently the most effective strategy that explore the evolution trajectory for cells. Future spatial transcriptomic techniques may enhance scRNA-seq’s spatial analysis. scRNA-seq reflects functional genomics and is pivotal for understanding cell fate, immune response dynamics, and cell migration.

At present, the main bottleneck in stroke treatment is improving patients’ functional recovery. Recovery strategies include surgery, pharmacological, and biological drug therapies. scRNA-seq provides insights into cell type-specific effects, off-target impacts, and heterogeneous responses, guiding drug candidate selection. In clinical development, it aids in biomarker identification for patient stratification, clarifies drug action/resistance mechanisms, and monitors drug responses/disease progression (Van de Sande et al., 2023). Reports on scRNA-seq based stroke treatments in clinical trials or with available drugs are scarce, limiting its use mainly to basic research. For instance, Kuikun Yang et al. developed a neutrophil membrane-coated nanoprodrug using fingolimod (FTY720) for post-stroke neuroinflammation, modulating the microglia gene Cebpb via scRNA-seq for anti-inflammatory effects (Zhao et al., 2024). In other neurological conditions, a *Cx3cr1* antagonist identified by scRNA-seq and spatial transcriptomics is in Phase II trials for depression. Epidural electrical stimulation of *Hoxa10* ^+^ neurons, located via scRNA-seq, sped walking recovery in paralyzed mice (Gong et al., 2025). Yangbao Miao et al. used scRNA-seq to devise a gut-brain axis drug delivery platform for central nervous system lymphoma (Gong et al., 2025).

In routine clinical biochemical testing, scRNA-seq has advanced in characterizing circulating blood cell clustering/differentiation across diseases (Fang et al., 2021). Studies focus on immune cells, emergent cell populations, molecular mechanisms, cell subset interactions, and tumor metastasis. However, experimental variations across settings, methods, and diseases require larger cohort validation. A routine scRNA-seq monitoring method is lacking (Fang et al., 2021). Yet, integrating public databases, artificial intelligence, and computational tech will boost scRNA-seq’s clinical use.

### 6.2 Disadvantage

scRNA-seq has limitations: First, in view of the methodology itself, scRNA-seq depends the random dissociation and capture of tissues or samples, which might result in the over- or under-expression of specific cell types. For example, neurons-distinguished by their large size and intricate synaptic architectures, usually are vulnerable to fragmentation during extraction, leading to capture challenges and data omission. Although current approaches for isolating neuronal nuclei could improve neuronal capture rates, they might unintentionally diminish the yield of other cell types. Furthermore, rare cell populations, such as stem cells or immune cell subsets constituting only a minor fraction of tumor tissues, are prone to significant loss during sample processing.

Second, results from scRNA-seq also have certain limitations, it cannot accurately determine cell type proportions, while the cells were identified based on the marker there is not necessarily only one marker to accurate cells. Moreover, scRNA-seq excels in tissue-specific cell type detection but struggles with dynamic immune responses, as immune cells migrate widely. Solutions for spatial orientation exist, but cellular metabolic dynamics remain challenging. Experimental dynamic monitoring is needed to deepen understanding of immune cell behavior post-ischemic stroke. ScRNA-seq isolates individual cells through tissue dissociation, but cells occupying distinct spatial niches within the original tissue often exhibit specialized functions. However, scRNA-seq disrupts the inherent spatial architecture of cells, leading to irreversible loss of spatial context. Contemporary strategies integrate scRNA-seq with spatial transcriptomic platforms like Visium HD and Xenium advanced technologies to simultaneously capture detailed gene expression profiles and precise spatial coordinates of single cells, thereby facilitating the accurate reconstruction of cellular spatial organization and corresponding gene expression patterns within intact tissues. Third, the price of scRNA-seq is still expensive at present (at around USD 1,500/per sample), and cell type identification and gene expression depend on database size. A higher database platform like (10×Genomics) could achieve the requests of larger amount of data analysis.

## 7 Future trend

### 7.1 Applied technological innovation

The rapid evolution of single-cell omics technologies has enabled unprecedented cellular resolution, crucial for constructing a human cell atlas. Future research will focus on multi-omics approaches, integrating scRNA-seq with chromatin accessibility, metabolome analysis, and high-resolution spatial transcriptomics. Two primary integration strategies: deconvolution and mapping. Deconvolution methods, like Seurat 3.0 and SpatialDWLS, dissect mixed mRNA transcripts into cellular subclusters (Longo et al., 2021). Mapping techniques, including LIGER, Seurat Integration, and Harmony, localize cells within tissues via RNA-seq. These advancements aid in understanding cellular biology, fostering targeted therapies and personalized medicine (Calvanese et al., 2022; Arora et al., 2023; Zhang et al., 2022; Moncada et al., 2020; Ji et al., 2020). Single-cell spatial transcriptomics, combined with next-gen sequencing and fluorescent labeling, will unravel biological mechanisms, enhanced by technologies like three-dimensional single-molecule fluorescence *in situ* hybridization (3D smFISH) and optical coherence tomography (OCT)/positron emission tomography (PET) fusions (Huang et al., 2024; Ye et al., 2024; Burger et al., 2021; Wang et al., 2022; Sun et al., 2022; Liu C. et al., 2023).

Moreover, the application of advanced algorithms, such as deep learning-based methods like STNet (Longo et al., 2021), XFuse, Saliency, Giotto, SpaOTsc, and novoSpaRc, promises to revolutionize our understanding of intact tissues (Wu et al., 2022; Yang et al., 2024). Spatial transcriptomic analyses offer insights into cell identities and tissue localizations, requiring tools like Seurat Integration, Harmony, and LIGER (Longo et al., 2021). Integrating data from various organ systems and diseases enriches understanding, offering potential for advanced therapeutic strategies (Longo et al., 2021; Moses and Pachter, 2022). Other analyses integrated technology to construct neurovascular unit organs or microfluidic chips simulating brain tissue metabolism (Spitzer et al., 2023). Peking University’s Zemin Zhang and Xianwen Ren developed the CSOmap model based on scRNA-seq, validated in melanoma and head/neck cancer models (Ren et al., 2020).

### 7.2 Applied application scope innovation

The core design of scRNA-seq in clinical disease research focuses on comparing pathological with healthy tissues to elucidate complex clinical theories. Limited patient samples, especially non-brain tumor tissues, hinder widespread clinical use. Exploring novel avenues, like focusing on shared mechanisms of rare diseases, is crucial. Research increasingly emphasizes innovative drug development, including repurposing. This technology supports rational therapeutic intervention design, identifying drugs targeting disrupted molecular pathways or modulating culprit cell types for precision medicine.

## 8 Conclusion

Single-cell sequencing technologies analyze entire tissues, revealing stroke as a systemic perturbation of cellular dynamics. Advances in single-cell sequencing technologies have surpassed traditional methods, promising deeper understanding of ischemic stroke’s cellular intricacies. Anticipating the future, enthusiasm is mounting for the expanded application of these technologies in ischemic stroke research, heralding the possibility of achieving a more profound insight into the disease’s cellular intricacies and underlying mechanisms.

## Glossary

Ackr3: Atypical chemokine receptor 3
Acta2: Actin alpha 2
*Adarb2*: Adenosine deaminase RNA specific B2
*ADP*: Adenosine diphosphate
*Aldoc*: Aldolase C
*Akt*: RAC-alpha serine/threonine-protein kinase
*Apoe*: Apolipoprotein E
*Arg1*: Arginase 1
*Ascl1*: Achaete scute complex like protein 1
BBB: Blood brain barrier
*Ccl2*: Chemokine ligand 2
*Ccl3*: Chemokine ligand 3
*Ccl4*: Chemokine ligand 4
*Cebpb*: CCAAT/Enhancer binding protein beta
*Ch25h*: Recombinant Cholesterol-25-hydroxylase
CNS: Central nervous system
*Creb*: cAMP-response element binding protein
CSOmap: Cellular spatial organization mapper
Ctss: Cathepsin S
*Cx3cl1*: C-X3-C motif chemokine ligand 1
*Cx3cr1*: C-X3-C motif chemokine receptor 1
*Cxcl12*: Chemokine CXCL12
*Dbp*: D-Box Binding PAR BZIP Transcription Factor
*Dnaja1*: DnaJ heat shock protein family member A1
Edu: 5-ethynyl-2'-deoxyuridine
ET-1: Endothelin 1
*ELISA*: Enzyme-linked immunosorbent assay
*Fabp5*: Fatty acid-binding protein 5
*Fcrls*: Fc receptor-like protein S
*Foxp3*: Forkhead box protein P3
*Gdf15*: Growth differentiation factor 15
Gpnmb: Human transmembrane glycoprotein NMB
*Gpr124*: G protein-coupled receptor 124
*Grm5*: Glutamate metabotropic receptor 5
*MHCII*: Major histocompatibility complex
H2-Aa: H-2 class II histocompatibility antigen, A alpha chain
H2-Ab1: H-2 class II histocompatibility antigen, A-D beta chain
*Hif1*: Hypoxia inducible factor-1
*Hif1a*: Hypoxia inducible factor-1 alpha
Hoxa10: Homeobox protein HOXA10
*Hsp90aa1*: Heat shock protein 90 alpha family class A member 1
*Hspa8*: Heat shock protein family A member 8
*Hsph1*: Heat shock protein family H member 1
*Nrg1*: Neuregulin 1
Nrg3: Neuregulin 3
Nrxn1: Neurexin-1
Nrxn3: Neurexin-3
Igf1: Insulin like growth factor 1
Ifn: Type I interferon
*Il1a*: Interleukin 1 alpha
*Il1b*: Interleukin 1beta
*Il6*: Interleukin 6
*Il10*: Interleukin 10
*Itgb2*: Integrin beta 2
*Itm2a*: Integral membrane protein 2a
*Jak*: Janus kinase
*Kcnip4*: Potassium voltage-gated channel-interacting protein 4
*Kcnq5*: Potassium voltage-gated channel subfamily Q member 5
*Lrg1*: Leucine-rich α2-glycoprotein 1
*Ly6c*: Lymphocyte antigen 6 complex
*Ly6c2*: Lymphocyte antigen 6 complex, locus C2
*Lyz2*: Lysozyme C-2
*MAPK*: Mitogen-activated protein kinase
*MCAO/R*: Middle cerebral artery occlusion/reperfusion
*MCAO*: Middle cerebral artery occlusion
*MHCII*: Major histocompatibility complex class II
*Mif*: Macrophage migration inhibitory factor
Nes: Nuclear export signal
NF-κB: NF-kappaB inhibitor alpha
NK cell: Natural killer cell
*Nrg1*: Neuregulin 1
Nrg3: Neuregulin 3
NRN: Neutrophil membrane camouflaged polyprodrug nanomedicine
Nrxn1: Neurexin 1
*Nrxn3*: Neurexin 3
*Oasl*: 2',5'-oligoadenylate synthetase like protein
*Olig2*: Oligodendrocyte line transcription factor 2
*Parp1*: Poly(ADP-ribose) polymerase 1
PCR: Polymerase chain reaction
Ptger4: Prostaglandin E receptor 4
Reck: Reversion-inducing cysteine-rich protein with Kazal motifs
RMB: Ren min bi
ROS: Reactive oxygen species
*Satb2*: Special AT-rich sequence-binding protein 2
Sema4C/G: SEMA4C antigen (recombinant protein)
scRNA-seq: Single-cell RNA sequencing
*Spp1*: Secreted phosphoprotein-1
Stat3: Signal transducer and activator of transcription 3
Stat5: Signal transducer and activator of transcription 5
Sult1a1: Sulfotransferase 1A1
Tgf-β: Transforming growth factor-beta
Tnf-α: Tumor necrosis factor-alpha
Tie2: Recombinant TEK tyrosine kinase, endothelial
*Tmem119*: Transmembrane protein 119
Vegf: Vascular endothelial growth factor
Vegfa: Vascular endothelial growth factor alpha
Wnt7a: Wingless-type MMTV integration site family, member 7A
Wnt7b: Wingless-type MMTV integration site family member 7B

## References

[B1] Amantea D. Micieli G. Tassorelli C. Cuartero M. I. Ballesteros I. Certo M. (2015). Rational modulation of the innate immune system for neuroprotection in ischemic stroke. Front. Neurosci. 9, 147. 10.3389/fnins.2015.00147 25972779 PMC4413676

[B2] Amin M. A. Volpert O. V. Woods J. M. Kumar P. Harlow L. A. Koch A. E. (2003). Migration inhibitory factor mediates angiogenesis via mitogen-activated protein kinase and phosphatidylinositol kinase. Circulation Res. 93, 321–329. 10.1161/01.RES.0000087641.56024.DA 12881477

[B3] Arora R. Cao C. Kumar M. Sinha S. Chanda A. McNeil R. (2023). Spatial transcriptomics reveals distinct and conserved tumor core and edge architectures that predict survival and targeted therapy response. Nat. Commun. 14, 5029. 10.1038/s41467-023-40271-4 37596273 PMC10439131

[B4] Bain J. M. Moore L. Ren Z. Simonishvili S. Levison S. W. (2013). Vascular endothelial growth factors A and C are induced in the SVZ following neonatal hypoxia-ischemia and exert different effects on neonatal glial progenitors. Transl. Stroke Res. 4, 158–170. 10.1007/s12975-012-0213-6 23565129 PMC3613784

[B5] Beuker C. Schafflick D. Strecker J.-K. Heming M. Li X. Wolbert J. (2022). Stroke induces disease-specific myeloid cells in the brain parenchyma and pia. Nat. Communications 13, 945. 10.1038/s41467-022-28593-1 35177618 PMC8854573

[B6] Bloom S. I. Islam M. T. Lesniewski L. A. Donato A. J. (2023). Mechanisms and consequences of endothelial cell senescence. Nat. Rev. Cardiol. 20, 38–51. 10.1038/s41569-022-00739-0 35853997 PMC10026597

[B7] Bormann D. Knoflach M. Poreba E. Riedl C. J. Testa G. Orset C. (2024). Single-nucleus RNA sequencing reveals glial cell type-specific responses to ischemic stroke in male rodents. Nat. Commun. 15, 6232. 10.1038/s41467-024-50465-z 39043661 PMC11266704

[B8] BRAIN Initiative Cell Census Network (BICCN) (2021). A multimodal cell census and atlas of the mammalian primary motor cortex. Nature 598, 86–102. 10.1038/s41586-021-03950-0 34616075 PMC8494634

[B9] Burger M. L. Cruz A. M. Crossland G. E. Gaglia G. Ritch C. C. Blatt S. E. (2021). Antigen dominance hierarchies shape TCF1+ progenitor CD8 T cell phenotypes in tumors. Cell 184, 4996–5014.e26. 10.1016/j.cell.2021.08.020 34534464 PMC8522630

[B10] Cai W. Hu M. Li C. Wu R. Lu D. Xie C. (2023). FOXP3+ macrophage represses acute ischemic stroke-induced neural inflammation. Autophagy 19, 1144–1163. 10.1080/15548627.2022.2116833 36170234 PMC10012925

[B11] Calvanese V. Capellera-Garcia S. Ma F. Fares I. Liebscher S. Ng E. S. (2022). Mapping human haematopoietic stem cells from haemogenic endothelium to birth. Nature 604, 534–540. 10.1038/s41586-022-04571-x 35418685 PMC9645817

[B12] Cao G.-Z. Hou J.-Y. Zhou R. Tian L.-L. Wang M.-L. Zhang Y. (2023). Single-cell RNA sequencing reveals that VIM and IFITM3 are vital targets of Dengzhan Shengmai capsule to protect against cerebral ischemic injury. J. Ethnopharmacol. 311, 116439. 10.1016/j.jep.2023.116439 37004745

[B13] Carter B. Zhao K. (2021). The epigenetic basis of cellular heterogeneity. Nat. Reviews. Genet. 22, 235–250. 10.1038/s41576-020-00300-0 33244170 PMC10880028

[B14] Chen X. Huang Y. Huang L. Huang Z. Hao Z.-Z. Xu L. (2024). A brain cell atlas integrating single-cell transcriptomes across human brain regions. Nat. Med. 30, 2679–2691. 10.1038/s41591-024-03150-z 39095595 PMC11405287

[B15] Chen D. Jiang H. Sun L. Nurzat Y. Qin H. Zhao Z. (2025). Neuron-targeted ROS-responsive liposomes for puerarin delivery remodel ischemic microenvironment via microglial modulation and neurovascular regeneration. J. Nanobiotechnology 23, 677. 10.1186/s12951-025-03730-2 41084040 PMC12519847

[B16] Cho Y.-E. Lee H. Bae H. R. Kim H. Yun S. Vorn R. (2022). Circulating immune cell landscape in patients who had mild ischaemic stroke. Stroke Vasc. Neurology 7, 319–327. 10.1136/svn-2021-001224 35264400 PMC9453838

[B17] Colombo E. Farina C. (2016). Astrocytes: key regulators of neuroinflammation. Trends Immunol. 37, 608–620. 10.1016/j.it.2016.06.006 27443914

[B18] Davidson S. Coles M. Thomas T. Kollias G. Ludewig B. Turley S. (2021). Fibroblasts as immune regulators in infection, inflammation and cancer. Nat. Rev. Immunol. 21, 704–717. 10.1038/s41577-021-00540-z 33911232

[B19] Del Águila Á. Zhang R. Yu X. Dang L. Xu F. Zhang J. (2024). Microglial heterogeneity in the ischemic stroke mouse brain of both sexes. Genome Med. 16, 95. 10.1186/s13073-024-01368-7 39095897 PMC11295600

[B20] Deng Y. Lu J. Sivakumar V. Ling E. A. Kaur C. (2008). Amoeboid microglia in the periventricular white matter induce oligodendrocyte damage through expression of proinflammatory cytokines via MAP kinase signaling pathway in hypoxic neonatal rats. Brain Pathol. Zurich, Switz. 18, 387–400. 10.1111/j.1750-3639.2008.00138.x 18371179 PMC8095524

[B21] Ewing-Crystal N. A. Mroz N. M. Larpthaveesarp A. Lizama C. O. Pennington R. Chiaranunt P. (2025). Dynamic fibroblast-immune interactions shape recovery after brain injury. Nature 646, 934–944. 10.1038/s41586-025-09449-2 40903576 PMC12545229

[B22] Fang H. Zeng Y. Zhang L. Chen C. Powell C. A. Wang X. (2021). Can single cell RNA sequencing reshape the clinical biochemistry of hematology: new clusters of circulating blood cells. Clin. Transl. Med. 11, e671. 10.1002/ctm2.671 34898038 PMC8666581

[B23] Faust T. E. Lee Y.-H. O’Connor C. D. Boyle M. A. Gunner G. Durán-Laforet V. (2025). Microglia-astrocyte crosstalk regulates synapse remodeling via Wnt signaling. Cell 188, 5212–5230.e21. 10.1016/j.cell.2025.08.023 40934914 PMC12489809

[B24] Frazier A. P. Mitchell D. N. Given K. S. Hunn G. Burch A. M. Childs C. R. (2023). Chronic changes in oligodendrocyte sub-populations after middle cerebral artery occlusion in neonatal mice. Glia 71, 1429–1450. 10.1002/glia.24349 36794545

[B25] Frosch M. Shimizu T. Wogram E. Amann L. Gruber L. Groisman A. I. (2025). Microglia-neuron crosstalk through Hex-GM2-MGL2 maintains brain homeostasis. Nature 646, 913–924. 10.1038/s41586-025-09477-y 40769205 PMC12545202

[B26] Garcia-Bonilla L. Shahanoor Z. Sciortino R. Nazarzoda O. Racchumi G. Iadecola C. (2024). Analysis of brain and blood single-cell transcriptomics in acute and subacute phases after experimental stroke. Nat. Immunol. 25, 357–370. 10.1038/s41590-023-01711-x 38177281

[B27] Gong Y. Zhou Z. Xu T. Liu F. Chen A. Zou L. (2025). Padlock-designed MOFs triggers an “avalanche effect” to enhance apoptosis and suppress metastasis in central nervous system lymphoma. Sci. Adv. 11, eadv2647. 10.1126/sciadv.adv2647 41042871 PMC12494020

[B28] Greenberg D. A. Jin K. (2013). Vascular endothelial growth factors (VEGFs) and stroke. Cell. Mol. Life Sci. CMLS 70, 1753–1761. 10.1007/s00018-013-1282-8 23475070 PMC3634892

[B29] Gullotta G. S. De Feo D. Friebel E. Semerano A. Scotti G. M. Bergamaschi A. (2023). Age-induced alterations of granulopoiesis generate atypical neutrophils that aggravate stroke pathology. Nat. Immunol. 24, 925–940. 10.1038/s41590-023-01505-1 37188941

[B30] Guo K. Luo J. Feng D. Wu L. Wang X. Xia L. (2021). Single-Cell RNA sequencing with combined use of bulk RNA sequencing to reveal cell heterogeneity and molecular changes at acute stage of ischemic stroke in mouse cortex penumbra area. Front. Cell Dev. Biol. 9, 624711. 10.3389/fcell.2021.624711 33692998 PMC7937629

[B31] Gupta N. Shyamasundar S. Patnala R. Karthikeyan A. Arumugam T. V. Ling E.-A. (2018). Recent progress in therapeutic strategies for microglia-mediated neuroinflammation in neuropathologies. Expert Opin. Ther. Targets 22, 765–781. 10.1080/14728222.2018.1515917 30138572

[B32] Han B. Zhou S. Zhang Y. Chen S. Xi W. Liu C. (2024). Integrating spatial and single-cell transcriptomics to characterize the molecular and cellular architecture of the ischemic mouse brain. Sci. Transl. Med. 16, eadg1323. 10.1126/scitranslmed.adg1323 38324639

[B33] Huang X. Cao Z. Qian J. Ding T. Wu Y. Zhang H. (2024). Nanoreceptors promote mutant p53 protein degradation by mimicking selective autophagy receptors. Nat. Nanotechnol. 19, 545–553. 10.1038/s41565-023-01562-5 38216684

[B34] Ji A. L. Rubin A. J. Thrane K. Jiang S. Reynolds D. L. Meyers R. M. (2020). Multimodal analysis of composition and spatial architecture in human squamous cell carcinoma. Cell 182, 497–514.e22. 10.1016/j.cell.2020.05.039 32579974 PMC7391009

[B35] Jin C. Shi Y. Shi L. Leak R. K. Zhang W. Chen K. (2023). Leveraging single-cell RNA sequencing to unravel the impact of aging on stroke recovery mechanisms in mice. Proc. Natl. Acad. Sci. U. S. A. 120, e2300012120. 10.1073/pnas.2300012120 37307473 PMC10288588

[B36] Jolivel V. Bicker F. Binamé F. Ploen R. Keller S. Gollan R. (2015). Perivascular microglia promote blood vessel disintegration in the ischemic penumbra. Acta Neuropathol. 129, 279–295. 10.1007/s00401-014-1372-1 25500713

[B37] Kent S. A. Miron V. E. (2024). Microglia regulation of central nervous system myelin health and regeneration. Nat. Rev. Immunol. 24, 49–63. 10.1038/s41577-023-00907-4 37452201

[B38] Kim S. Lee W. Jo H. Sonn S.-K. Jeong S.-J. Seo S. (2022). The antioxidant enzyme Peroxiredoxin-1 controls stroke-associated microglia against acute ischemic stroke. Redox Biol. 54, 102347. 10.1016/j.redox.2022.102347 35688114 PMC9184746

[B39] Kim G. S. Harmon E. Gutierrez M. Stephenson J. Chauhan A. Banerjee A. (2023). Single-cell analysis identifies Ifi27l2a as a novel gene regulator of microglial inflammation in the context of aging and stroke. Res. Square. 10.21203/rs.3.rs-2557290/v1 39953063 PMC11828888

[B40] Kiss T. Nyúl-Tóth Á. Balasubramanian P. Tarantini S. Ahire C. DelFavero J. (2020). Single-cell RNA sequencing identifies senescent cerebromicrovascular endothelial cells in the aged mouse brain. Geroscience 42, 429–444. 10.1007/s11357-020-00177-1 32236824 PMC7205992

[B41] Kriz J. Lalancette-Hébert M. (2009). Inflammation, plasticity and real-time imaging after cerebral ischemia. Acta Neuropathol. 117, 497–509. 10.1007/s00401-009-0496-1 19225790

[B42] Kwak D. Park J. H. Kim Y. H. Yoo H. I. (2025). Decoding hippocampal subfield and glial responses in ischemia using single-cell transcriptomics. J. Transl. Med. 23, 671. 10.1186/s12967-025-06738-2 40528218 PMC12175379

[B43] Lauro C. Chece G. Monaco L. Antonangeli F. Peruzzi G. Rinaldo S. (2019). Fractalkine modulates microglia metabolism in brain ischemia. Front. Cell. Neurosci. 13, 414. 10.3389/fncel.2019.00414 31607865 PMC6755341

[B44] Li Z. Song Y. He T. Wen R. Li Y. Chen T. (2021). M2 microglial small extracellular vesicles reduce glial scar formation via the miR-124/STAT3 pathway after ischemic stroke in mice. Theranostics 11, 1232–1248. 10.7150/thno.48761 33391532 PMC7738903

[B45] Li X. Lyu J. Li R. Jain V. Shen Y. Del Águila Á. (2022). Single-cell transcriptomic analysis of the immune cell landscape in the aged mouse brain after ischemic stroke. J. Neuroinflammation 19, 83. 10.1186/s12974-022-02447-5 35392936 PMC8988369

[B46] Li H. Liu P. Zhang B. Yuan Z. Guo M. Zou X. (2023). Acute ischemia induces spatially and transcriptionally distinct microglial subclusters. Genome Med. 15, 109. 10.1186/s13073-023-01257-5 38082331 PMC10712107

[B47] Liddelow S. A. Barres B. A. (2017). Reactive astrocytes: production, function, and therapeutic potential. Immunity 46, 957–967. 10.1016/j.immuni.2017.06.006 28636962

[B48] Liddelow S. A. Guttenplan K. A. Clarke L. E. Bennett F. C. Bohlen C. J. Schirmer L. (2017). Neurotoxic reactive astrocytes are induced by activated microglia. Nature 541, 481–487. 10.1038/nature21029 28099414 PMC5404890

[B49] Liu W. Qi Z. Li W. Liang J. Zhao L. Shi Y. (2022). M1 microglia induced neuronal injury on ischemic stroke via mitochondrial crosstalk between microglia and neurons. Oxidative Med. Cell. Longev. 2022, 4335272. 10.1155/2022/4335272 36478988 PMC9722306

[B50] Liu P.-Y. Li H.-Q. Dong M.-Q. Gu X.-Y. Xu S.-Y. Xia S.-N. (2023a). Infiltrating myeloid cell-derived properdin markedly promotes microglia-mediated neuroinflammation after ischemic stroke. J. Neuroinflammation 20, 260. 10.1186/s12974-023-02946-z 37951917 PMC10640761

[B51] Liu C. Zhang M. Yan X. Ni Y. Gong Y. Wang C. (2023b). Single-cell dissection of cellular and molecular features underlying human cervical squamous cell carcinoma initiation and progression. Sci. Adv. 9, eadd8977. 10.1126/sciadv.add8977 36706185 PMC9882988

[B52] Liu F. Cheng X. Zhao C. Zhang X. Liu C. Zhong S. (2024). Single-cell mapping of brain myeloid cell subsets reveals key transcriptomic changes favoring neuroplasticity after ischemic stroke. Neurosci. Bull. 40, 65–78. 10.1007/s12264-023-01109-7 37755676 PMC10774469

[B53] Liu Y. Lin W. Bai Z. Ge Y. Xiao Y. Zhu F. (2025). Lcn2 from neutrophil extracellular traps induces astrogliosis and post-stroke emotional disorders. Neuron 113 (24), 4199–4216. 10.1016/j.neuron.2025.09.018 41075784

[B54] Longo S. K. Guo M. G. Ji A. L. Khavari P. A. (2021). Integrating single-cell and spatial transcriptomics to elucidate intercellular tissue dynamics. Nat. Rev. Genet. 22, 627–644. 10.1038/s41576-021-00370-8 34145435 PMC9888017

[B55] Ma H. Li H. Zhang Y. Zhou Y. Liu H. Xu H. (2023). Microglia exhibit distinct heterogeneity rather than M1/M2 polarization within the early stage of acute ischemic stroke. Aging Dis. 14, 2284–2302. 10.14336/AD.2023.0505 37199734 PMC10676790

[B56] Ma Y. Wang J. Wang Y. Yang G.-Y. (2017). The biphasic function of microglia in ischemic stroke. Prog. Neurobiol. 157, 247–272. 10.1016/j.pneurobio.2016.01.005 26851161

[B57] Marin M. A. Carmichael S. T. (2019). Mechanisms of demyelination and remyelination in the young and aged brain following white matter stroke. Neurobiol. Dis. 126, 5–12. 10.1016/j.nbd.2018.07.023 30031782

[B58] Martin M. Vermeiren S. Bostaille N. Eubelen M. Spitzer D. Vermeersch M. (2022). Engineered Wnt ligands enable blood-brain barrier repair in neurological disorders. Science 375, eabm4459. 10.1126/science.abm4459 35175798

[B59] McTigue D. M. Tripathi R. B. (2008). The life, death, and replacement of oligodendrocytes in the adult CNS. J. Neurochem. 107, 1–19. 10.1111/j.1471-4159.2008.05570.x 18643793

[B60] Miron V. E. Boyd A. Zhao J.-W. Yuen T. J. Ruckh J. M. Shadrach J. L. (2013). M2 microglia and macrophages drive oligodendrocyte differentiation during CNS remyelination. Nat. Neurosci. 16, 1211–1218. 10.1038/nn.3469 23872599 PMC3977045

[B61] Moncada R. Barkley D. Wagner F. Chiodin M. Devlin J. C. Baron M. (2020). Integrating microarray-based spatial transcriptomics and single-cell RNA-seq reveals tissue architecture in pancreatic ductal adenocarcinomas. Nat. Biotechnol. 38, 333–342. 10.1038/s41587-019-0392-8 31932730

[B62] Moses L. Pachter L. (2022). Museum of spatial transcriptomics. Nat. Methods 19, 534–546. 10.1038/s41592-022-01409-2 35273392

[B63] Muñoz-Castañeda R. Zingg B. Matho K. S. Chen X. Wang Q. Foster N. N. (2021). Cellular anatomy of the mouse primary motor cortex. Nature 598, 159–166. 10.1038/s41586-021-03970-w 34616071 PMC8494646

[B64] Nakamura A. Sakai S. Taketomi Y. Tsuyama J. Miki Y. Hara Y. (2023). PLA2G2E-mediated lipid metabolism triggers brain-autonomous neural repair after ischemic stroke. Neuron 111, 2995–3010.e9. 10.1016/j.neuron.2023.06.024 37490917

[B65] Norden D. M. Fenn A. M. Dugan A. Godbout J. P. (2014). TGFβ produced by IL-10 redirected astrocytes attenuates microglial activation. Glia 62, 881–895. 10.1002/glia.22647 24616125 PMC4061706

[B66] Patir A. Barrington J. Szymkowiak S. Brezzo G. Straus D. Alfieri A. (2024). Phenotypic and spatial heterogeneity of brain myeloid cells after stroke is associated with cell ontogeny, tissue damage, and brain connectivity. Cell Rep. 43, 114250. 10.1016/j.celrep.2024.114250 38762882

[B67] Qi L. Wang C. Deng L. Pan J.-J. Suo Q. Wu S. (2024). Low-intensity focused ultrasound stimulation promotes stroke recovery via astrocytic HMGB1 and CAMK2N1 in mice. Stroke Vasc. Neurol. 9, 505–518. 10.1136/svn-2023-002614 38191183 PMC11732924

[B68] Qiu M. Zong J.-B. He Q.-W. Liu Y.-X. Wan Y. Li M. (2022). Cell heterogeneity uncovered by single-cell RNA sequencing offers potential therapeutic targets for ischemic stroke. Aging Dis. 13, 1436–1454. 10.14336/AD.2022.0212 36186129 PMC9466965

[B69] Ren X. Zhong G. Zhang Q. Zhang L. Sun Y. Zhang Z. (2020). Reconstruction of cell spatial organization from single-cell RNA sequencing data based on ligand-receptor mediated self-assembly. Cell Res. 30, 763–778. 10.1038/s41422-020-0353-2 32541867 PMC7608415

[B70] Ren X. Zhang L. Zhang Y. Li Z. Siemers N. Zhang Z. (2021). Insights gained from single-cell analysis of immune cells in the tumor microenvironment. Annu. Rev. Immunol. 39, 583–609. 10.1146/annurev-immunol-110519-071134 33637019

[B71] Revuelta M. Elicegui A. Moreno-Cugnon L. Bührer C. Matheu A. Schmitz T. (2019). Ischemic stroke in neonatal and adult astrocytes. Mech. Ageing Dev. 183, 111147. 10.1016/j.mad.2019.111147 31493435

[B72] Roth M. Carlsson R. Buizza C. Enström A. Paul G. (2025). Pericyte response to ischemic stroke precedes endothelial cell death and blood-brain barrier breakdown. J. Cereb. Blood Flow. Metab. 45, 617–629. 10.1177/0271678X241261946 39053491 PMC11571979

[B73] Ruan Z. Cao G. Qian Y. Fu L. Hu J. Xu T. (2023). Single-cell RNA sequencing unveils Lrg1’s role in cerebral ischemia‒reperfusion injury by modulating various cells. J. Neuroinflammation 20, 285. 10.1186/s12974-023-02941-4 38037097 PMC10687904

[B74] Schmidt-Pogoda A. Ruck T. Strecker J. K. Hoppen M. Fazio L. Vinnenberg L. (2025). Exercise facilitates post-stroke recovery through mitigation of neuronal hyperexcitability via interleukin-10 signaling. Nat. Commun. 16, 8928. 10.1038/s41467-025-62631-y 41062473 PMC12508215

[B75] Schwartz M. (2003). Macrophages and microglia in central nervous system injury: are they helpful or harmful? J. Cereb. Blood Flow Metabolism Official Journal Int. Soc. Cereb. Blood Flow Metabolism 23, 385–394. 10.1097/01.WCB.0000061881.75234.5E 12679714

[B76] Scott E. Y. Safarian N. Casasbuenas D. L. Dryden M. Tockovska T. Ali S. (2024). Integrating single-cell and spatially resolved transcriptomic strategies to survey the astrocyte response to stroke in male mice. Nat. Commun. 15, 1584. 10.1038/s41467-024-45821-y 38383565 PMC10882052

[B77] Sheng Y. Duan X. Liu Y. Li F. Ma S. Shang X. (2021). Tie2-expressing monocytes/macrophages promote cerebral revascularization in peri-infarct lesions upon ischemic insult. Signal Transduct. Target Ther. 6, 295. 10.1038/s41392-021-00637-w 34366430 PMC8349906

[B78] Shi L. Sun Z. Su W. Xu F. Xie D. Zhang Q. (2021). Treg cell-derived osteopontin promotes microglia-mediated white matter repair after ischemic stroke. Immunity 54, 1527–1542.e8. 10.1016/j.immuni.2021.04.022 34015256 PMC8282725

[B79] Spitzer D. Khel M. I. Pütz T. Zinke J. Jia X. Sommer K. (2023). A flow cytometry-based protocol for syngenic isolation of neurovascular unit cells from mouse and human tissues. Nat. Protoc. 18, 1510–1542. 10.1038/s41596-023-00805-y 36859615

[B80] Sun K. Xu R. Ma F. Yang N. Li Y. Sun X. (2022). scRNA-seq of gastric tumor shows complex intercellular interaction with an alternative T cell exhaustion trajectory. Nat. Commun. 13, 4943. 10.1038/s41467-022-32627-z 35999201 PMC9399107

[B81] Tei N. Tanaka J. Sugimoto K. Nishihara T. Nishioka R. Takahashi H. (2013). Expression of MCP-1 and fractalkine on endothelial cells and astrocytes may contribute to the invasion and migration of brain macrophages in ischemic rat brain lesions. J. Neurosci. Res. 91, 681–693. 10.1002/jnr.23202 23400803

[B82] Tripp H. F. Lewis W. Veronee C. Damiano R. J. German L. D. Lowe J. E. (1986). Effects of acute tachycardia on left ventricular adenine nucleotide levels and subsequent tolerance to ischemia. J. Thorac. Cardiovasc Surg. 92, 931–935. 3773548

[B83] Van de Sande B. Lee J. S. Mutasa-Gottgens E. Naughton B. Bacon W. Manning J. (2023). Applications of single-cell RNA sequencing in drug discovery and development. Nat. Rev. Drug Discov. 22, 496–520. 10.1038/s41573-023-00688-4 37117846 PMC10141847

[B84] Wang C. Yu Q. Song T. Wang Z. Song L. Yang Y. (2022). The heterogeneous immune landscape between lung adenocarcinoma and squamous carcinoma revealed by single-cell RNA sequencing. Signal Transduct. Target Ther. 7, 289. 10.1038/s41392-022-01130-8 36008393 PMC9411197

[B85] Wang R. Li H. Ling C. Zhang X. Lu J. Luan W. (2023). A novel phenotype of B cells associated with enhanced phagocytic capability and chemotactic function after ischemic stroke. Neural Regen. Res. 18, 2413–2423. 10.4103/1673-5374.371365 37282471 PMC10360087

[B86] Wu Y. Cheng Y. Wang X. Fan J. Gao Q. (2022). Spatial omics: navigating to the golden era of cancer research. Clin. Transl. Med. 12, e696. 10.1002/ctm2.696 35040595 PMC8764875

[B87] Wu Y. Xie L. Sun J. Wang Q. Xia W. Cai Q. (2025). Response of astrocytes and their interaction with surrounding brain cells after acute ischemia-reperfusion analyzed by single-cell transcriptome sequencing. Brain Res. Bull. 226, 111355. 10.1016/j.brainresbull.2025.111355 40286940

[B88] Xie X. P. Laks D. R. Sun D. Poran A. Laughney A. M. Wang Z. (2020). High-resolution mouse subventricular zone stem-cell niche transcriptome reveals features of lineage, anatomy, and aging. Proc. Natl. Acad. Sci. U. S. A. 117, 31448–31458. 10.1073/pnas.2014389117 33229571 PMC7733854

[B89] Xie L. Zhang S. Huang L. Peng Z. Lu H. He Q. (2023). Single-cell RNA sequencing of peripheral blood reveals that monocytes with high cathepsin S expression aggravate cerebral ischemia-reperfusion injury. Brain, Behav. Immun. 107, 330–344. 10.1016/j.bbi.2022.11.001 36371010

[B90] Yang W. Wang P. Xu S. Wang T. Luo M. Cai Y. (2024). Deciphering cell-cell communication at single-cell resolution for spatial transcriptomics with subgraph-based graph attention network. Nat. Commun. 15, 7101. 10.1038/s41467-024-51329-2 39155292 PMC11330978

[B91] Yao Z. van Velthoven C. T. J. Kunst M. Zhang M. McMillen D. Lee C. (2023). A high-resolution transcriptomic and spatial atlas of cell types in the whole mouse brain. Nature 624, 317–332. 10.1038/s41586-023-06812-z 38092916 PMC10719114

[B92] Ye Q.-N. Zhu L. Liang J. Zhao D.-K. Tian T.-Y. Fan Y.-N. (2024). Orchestrating NK and T cells via tri-specific nano-antibodies for synergistic antitumor immunity. Nat. Commun. 15, 6211. 10.1038/s41467-024-50474-y 39043643 PMC11266419

[B93] Yin Y. Hu J. Wu H. Yang X. Qi J. Huang L. (2025). Astrocytic dopamine D1 receptor modulates glutamatergic transmission and synaptic plasticity in the prefrontal cortex through d-serine. Acta Pharm. Sin. B 15, 4692–4710. 10.1016/j.apsb.2025.07.034 41049738 PMC12491719

[B94] Zeng F. Cao J. Hong Z. Liu Y. Hao J. Qin Z. (2023). Single-cell analyses reveal the dynamic functions of Itgb2+ microglia subclusters at different stages of cerebral ischemia-reperfusion injury in transient middle cerebral occlusion mice model. Front. Immunol. 14, 1114663. 10.3389/fimmu.2023.1114663 37063847 PMC10098327

[B95] Zhan R. Meng X. Tian D. Xu J. Cui H. Yang J. (2023). NAD+ rescues aging-induced blood-brain barrier damage via the CX43-PARP1 axis. Neuron 111, 3634–3649.e7. 10.1016/j.neuron.2023.08.010 37683629

[B96] Zhang Q. He Y. Luo N. Patel S. J. Han Y. Gao R. (2019). Landscape and dynamics of single immune cells in hepatocellular carcinoma. Cell 179, 829–845.e20. 10.1016/j.cell.2019.10.003 31675496

[B97] Zhang Q. Abdo R. Iosef C. Kaneko T. Cecchini M. Han V. K. (2022). The spatial transcriptomic landscape of non-small cell lung cancer brain metastasis. Nat. Commun. 13, 5983. 10.1038/s41467-022-33365-y 36216799 PMC9551067

[B98] Zhang Q. Shi S. Tang Y. Qu C. Wen S. Pan Y. (2023a). Manf enhances the pyroptosis inhibition of bone Marrow-derived mesenchymal stem cells to relieve cerebral infarction injury. Neuroscience 510, 109–128. 10.1016/j.neuroscience.2022.11.002 36529294

[B99] Zhang Y. Guo Y. Li R. Huang T. Li Y. Xie W. (2023b). Novel CH25H+ and OASL+ microglia subclusters play distinct roles in cerebral ischemic stroke. J. Neuroinflammation 20, 115. 10.1186/s12974-023-02799-6 37183260 PMC10184422

[B100] Zhang W. Wang X. Zhang B. Yi M. Lu Y. Wang S. (2025). Single-cell RNA-seq revealed the immune microenvironment reprogramming by dexmedetomidine treatment in ischemic stroke. Mol. Neurobiol. 62, 16150–16167. 10.1007/s12035-025-05237-1 40751033 PMC12559068

[B101] Zhao Y. Xiao C. Chen H. Zhu R. Zhang M. Liu H. (2023). Single-cell RNA-seq reveals changes in cell subsets in the cortical microenvironment during acute phase of ischemic stroke rats. J. Integr. Neurosci. 22, 128. 10.31083/j.jin2205128 37735120

[B102] Zhao Y. Li Q. Niu J. Guo E. Zhao C. Zhang J. (2024). Neutrophil membrane-camouflaged polyprodrug nanomedicine for inflammation suppression in ischemic stroke therapy. Adv. Mater. Deerf. Beach, Fla. 36, e2311803. 10.1002/adma.202311803 38519052

[B103] Zhao L. Chen Y. Ding X. Li H. Li J. (2025). Targeting *Atf4* for enhanced neuroprotection: role of quercetin-loaded EVs in ischemic stroke. J. Pharm. Anal. 15, 101312. 10.1016/j.jpha.2025.101312 41079784 PMC12509756

[B104] Zheng Y. He R. Wang P. Shi Y. Zhao L. Liang J. (2019). Exosomes from LPS-stimulated macrophages induce neuroprotection and functional improvement after ischemic stroke by modulating microglial polarization. Biomaterials Sci. 7, 2037–2049. 10.1039/c8bm01449c 30843911

[B105] Zheng K. Lin L. Jiang W. Chen L. Zhang X. Zhang Q. (2022). Single-cell RNA-seq reveals the transcriptional landscape in ischemic stroke. J. Cereb. Blood Flow Metabolism Official Journal Int. Soc. Cereb. Blood Flow Metabolism 42, 56–73. 10.1177/0271678X211026770 34496660 PMC8721774

[B106] Zheng Y. Ren Z. Liu Y. Yan J. Chen C. He Y. (2025a). T cell interactions with microglia in immune-inflammatory processes of ischemic stroke. Neural Regen. Res. 20, 1277–1292. 10.4103/NRR.NRR-D-23-01385 39075894 PMC11624874

[B107] Zheng H. Feng Y. Tang J. Yu F. Wang Z. Xu J. (2025b). Astrocyte-secreted cues promote neural maturation and augment activity in human forebrain organoids. Nat. Commun. 16, 2845. 10.1038/s41467-025-58295-3 40122897 PMC11930946

[B108] Zhou J. Zhang L. Peng J. Zhang X. Zhang F. Wu Y. (2024). Astrocytic LRP1 enables mitochondria transfer to neurons and mitigates brain ischemic stroke by suppressing ARF1 lactylation. Cell Metab. 36, 2054–2068.e14. 10.1016/j.cmet.2024.05.016 38906140

[B109] Zucha D. Abaffy P. Kirdajova D. Jirak D. Kubista M. Anderova M. (2024). Spatiotemporal transcriptomic map of glial cell response in a mouse model of acute brain ischemia. Proc. Natl. Acad. Sci. U. S. A. 121, e2404203121. 10.1073/pnas.2404203121 39499634 PMC11573666

