# Supplementary material for: Insights gained from single-cell sequencing analysis of ischemic stroke
Source: Front Cell Dev Biol. 2026 Apr 21;14:1784660. doi: 10.3389/fcell.2026.1784660 (PMC13139180; doi:10.3389/fcell.2026.1784660)
Supplement: Supplementary file 1 [file Supplementaryfile1.doc]

**Table 2 Biomarkers and functional characteristics of cell heterogeneity responsible for core functions**

| **Cell type** | **Biomarkers of key cell subsets** | **Description** | **Model** | **Species** | **Platform** |
| --- | --- | --- | --- | --- | --- |
| Microglia | *Ch25h* | *Ch25h⁺* microglia showed heightened phagocytosis and neuroprotection post-stroke | MCAO 1 h and reperfusion 3 d | Mice | Illumina sequencer |
| Microglia | *Oasl* | *Oasl⁺* subcluster accumulated in ischemic brain post-stroke, linked to neuroinflammation progression, worsening in aged mice brains | MCAO 1 h and reperfusion 3 d | Mice | Illumina sequencer |
| Microglia | *Gpr34, Olfml3, P2ry12, Tmem119, Selplg, Siglech* | *Gpr34, Olfml3, P2ry12, Tmem119, Selplg,* and *Siglech* had relatively low expression in highly inflammatory ischemic injury-associated microglial subclusters | MCAO 24 h | Mice | 10×Genomics |
| Microglia | *Mmp12, Adam8* | High *Mmp12* expression in microglial_2 cluster damages BBB post-ischemic stroke; exclusively upregulated *Adam8* in it may modulate microglia-mediated neuroprotection | MCAO 24 h | Mice | 10×Genomics |
| Microglia | *Gadd45b, Eif4ebp1, Zbtb16* | *Gadd45b, Eif4ebp1,* and *Zbtb16* showed specific enrichment in the MCAO group | MCAO 1 h and reperfusion 24 h | Mice | 10×Genomics |
| Microglia | *Cxcr2, S100a8, Il1b, Mmp9* | *Cxcr2, S100a8, Il1b,* and *Mmp9* with "neutrophil-like" profile indicate stroke-specific microglia state in aged brain post-stroke | MCAO 6 h and reperfusion 1 d, 3 d, and 7 d | Mice | 10×Genomics |
| Microglia | *Prdx1, Txn1, Srx1, Mt1, Mt2* | *Prdx1*, an antioxidant enzyme in stroke-associated microglia, mediates ROS defense genes (including *Txn1, Srx1, Mt1, and Mt2*) highlighting its necessity for microglial activation and antioxidant role | MCAO 24 h | Mice | BD Biosciences |
| Microglia | *Itgb2* | *Itgb2^+^* microglial subcluster was mainly involved in energy metabolism, cell cycle, angiogenesis, and neuronal myelin formation/repair at 1, 3, and 7 d post-MCAO | MCAO 1.5 h and reperfusion 1, 3, and 7 d | Mice | Bioanalyzer 2100 |
| Microglia | *Spp1, Msr1, Lgals3, Ccl2, Ccl12, Top2A, Mki67, Stmn1, Apoe, Cst7, Clec7a, Lyz2, Spp1, Igf1, Lgals3bp, Apoc1, Lpl, Gpnmb, Itgax, Axl, Itgax,Il1b, Nfkbiz, Cd83, Ccl4* | Microglia_4 subcluster highly expressed genes related to damaged cell clearance, tissue repair (*Spp1, Msr1, Lgals3*), and chemokines (*Ccl2, Ccl12*). Microglia_5 expressed mitotic genes (*Top2A, Mki67, Stmn1*), indicating proliferation during acute stroke. Microglia_3, 4, 7 were defined by disease-associated microglia genes (*Apoe, Cst7, Clec7a, Lyz2, Spp1, Igf1, Lgals3bp, Apoc1, Lpl, Gpnmb, Itgax*) and neurodegeneration-related genes (*Gpnmb, Axl, Itgax*). Microglia_6 upregulated immune genes (*Il1b, Nfkbiz, Cd83, Ccl4*) | MCAO 35 min and reperfusion 2 and 4 d | Mice | Illumina NextSeq500 |
| Microglia | *Il1a, Il1b, Il6, Il18, Tnf, Hmox1, Ptgs2, Ccl2, Ccl3, Ccl4, Ccl5, Cxcl2, Cxcl16* | Increased expression of pro-inflammatory and inflammation-responsive genes (*Il1a, Il1b, Il6, Il18, Tnf, Hmox1, Ptgs2*) was identified in ischemic core-associated microglia compared to other subclusters. Specifically, chemokine genes (*Ccl2, Ccl3, Ccl4, Ccl5, Cxcl2, Cxcl16*) showed higher expression in ischemic core-associated microglia than in ischemic penumbra-associated microglia | MCAO 1 h and reperfusion 3, 12, 24, or 72 h | Mice | 10×Genomics |
| Microglia | *P2ry12, Fcrls, Hexb, Tmem119, Gpr34, Cst7, Spp1, Apoe, Lgals3, Ccl3, Ccl4, Egr1, Il1b, Tnf* | Heterogeneity of reactive Microglia_3 days post-MCAO was explored by subclustering *P2ry12^+^, Fcrls^+^* cells. Microglia_4 expressed homeostatic microglial genes (*Hexb, Tmem119, Gpr34, P2ry12*), indicative of a homeostatic state. Microglia_3, 7 and 16, derived exclusively from MCAO samples, exhibited high expression genes (*Cst7, Spp1, Apoe, Lgals3*), resembling disease-associated microglia. Microglia_10 and 16 expressed chemokine/cytokine genes (*Ccl3, Ccl4, Egr1, Il1b, Tnf*) | 3 d after permanent distal middle cerebral  artery occlusion | Mice | 10× Genomics |
| Microglia | *Cd45* | Seven stroke-linked microglial clusters were found, with macrophage-like, IFN-responsive subclusters having upregulated *Cd45* being the most dominant in post-stroke brains | Permanent middle cerebral artery occlusion | Mice | 10× Genomics |
| Microglia | *Cebpb, Nlpr3, Cxcl2, Pgk1, Ctsb, Zeb1, Klf2,* | Following cerebral ischemia-reperfusion, microglial subclusters undergo differentiation, shifting from microglia_1 and microglia_2 to microglia_3 and microglia_4, accompanied by an upregulation in the expression of *Cebpb, Nlpr3, Cxcl2, Pgk1,* and *Ctsb,* alongside a downregulation of *Zeb1* and *Klf2,* thereby facilitating neuroinflammation subsequent to ischemic stroke | MCAO 1 h and reperfusion 24 h | Mice | 10×Genomics |
| Microglia | *Fcrl2, Cd68, CD74, Nfkb1, Ox-6, Pnn, Zfhx3, P2rx7* | Under 4-vessel occlusion in the hippocampal CA1 and CA3-DG, the CA1 region shows a predominance of pro-inflammatory microglia cluster 1 with upregulated *Fcrl2, CD68, CD74, Nfkb1,* and *Ox-6.* In sham-operation group, homeostatic microglia express Tmem119 and neuroprotective genes *Pnn, Zfhx3,* and *P2rx7* | Four vessel occlusion surgery | Rat | 10×Genomics |
| Microglia | *Mki67, Mif, Mertk* | Within the MCAO group, microglial subclusters 4 and 7 displayed pronounced expression of the proliferation marker *Mki67*, whereas subcluster 2 exhibited heightened expression of *Mif,* indicative of severe metabolic irregularities. Furthermore, subclusters 0, 1, 3, 4, 5, and 6 expressed the phagocytic marker *Mertk*, suggesting that these microglia retained their phagocytic capabilities | MCAO 1.5 h and reperfusion 24 h | Mice | 10×Genomics |
| Macrophage | *H2-Aa, H2-Ab1 and CD74, Cox7b, Cox8a, Uqcr11* | Macrophages from the MCAO group exhibited higher levels of MHCII-related antigen presentation molecules (*H2-Aa, H2-Ab1, CD74*). A distinct MCAO-derived cluster was characterized by oxidative phosphorylation and respiratory electron transport chain, expressing *Cox7b, Cox8a,* and *Uqcr11* | MCAO 24 h | Mice | 10×Genomics |
| Macrophage | *Apoe, Arg1, Ym1, Cd93, Hmox1, Tgfbi, Hif1a, Cybb, Il1b* | The dominant monocyte/macrophage cluster increas in the post-stroke brain is macrophage_1 subcluster, characteristically express several potentially protective genes including *Apoe, Arg1, Ym1, Cd93, Hmox1, Tgfbi,* and *Hif1a*, while macrophage_1 subcluster express *Cybb* and *Il1b*, suggesting that most monocyte/macrophages could be beneficial on post-stroke day 3 | MCAO 6 h and reperfusion 1, 3 , and 7 d | Mice | 10×Genomics |
| Macrophage | I*L1b, Cxcl2, Ifitm1, Srgn, Hmox1, S100a9, S100a8, Mmp9, Mmp8, Hpgd, Ctsc, Ccl2, Maf, Stab1* | Compared to the sham group, the MCAO group showed upregulation of *IL1b, Cxcl2, Ifitm1, Srgn, Hmox1, S100a9, S100a8, Mmp9, Mmp8* genes and downregulation of *Hpgd, Ctsc, Ccl2, Maf, Stab1* genes | MCAO 1 h and reperfusion 24 h | Mice | 10×Genomics |
| Macrophage | *Foxp3* | *Foxp3^+^* macrophages, distinct from M1/M2 subsets, exhibit superactive efferocytosis. Foxp3 enhances macrophage phagocytosis via cargo metabolism and characteristic with heightened scavenging ability | MCAO 1 h and reperfusion 5 d | Mice | Novaseq 6000 |
| Macrophage | *Ly6c2, Ms4a4c, Adgre1, Ifitm3, CD74, H2-Aa, H2-Ab1, H2-Eb1, Ccl2, Ccrl2, Ccl4, Cxcl2, Ccl3, Arg1, F13a1, Gas6, Cd63, Nrp1* | Macrophages were divided into 4 subtypes, including intermediate state of monocyte and macrophage (*Ly6c2 and Ms4a4c, Adgre1,* and *Ifitm3*), high MHC II macrophage (*CD74, H2-Aa, H2-Ab1,* and *H2-Eb1*), chemokine-enriched macrophage (*Ccl2, Ccrl2, Ccl4, Cxcl2,* and *Ccl3*), and *Arg1* high-expressed macrophage (*Arg1, F13a1, Gas6, Cd63,* and *Nrp1*) | Photothrombosis of cortical microvessels 5 min and 3 d after surgery | Mice | 10×Genomics |
|  |  |  |  |  |  |
| Oligodendrocyte | *Olig, Edu* | *Olig2^+^*, *Edu^+^* cells rose in ipsilateral striatum 14 d post-MCAO, then declined by 28 d | MCAO 1 h and reperfusion 14 and 28-30 d | Mice | 10×Genomics |
| Oligodendrocyte | *Sgk3* | *Sgk3* within oligodendrocytes may exhibit a subcluster-specific role in governing cell fate during the early stages of ischemic stroke | MCAO 1 h and reperfusion 24 h | Mice | 10×Genomics |
| Oligodendrocyte | *Nrxn1, Nrxn3, Nrg1, Nrg3, Kcnip4, Grm5, Kcnq5* | Differentially expressed genes downregulated in the oligodendrocyte subcluster from the MCAO contralateral side compared to the sham-operated group encompass neurexins and neuregulins (*Nrxn1, Nrxn3, Nrg1, Nrg3*), along with genes encoding neurotransmitter receptors, ion channels, and ion channel-interacting proteins (*Kcnip4, Grm5, Kcnq5*) | Permanent middle cerebral artery occlusion model in rats and thromboembolic stroke model in mice | Rats and mice | NovaSeq 6000 |
| Oligodendrocyte | *Cd44, Vim, Il33, Runx1, Runx2, Vim,* *Il33, Ccnf, Vgf, Fgf2* | Canonical pan-reactive astrocyte markers *Cd44, Vim, Il33, Runx1, Runx2,* and the neuroprotective alarmin were upregulated in oligodendrocyte_1 subcluster, with *Vim* and *Il33* previously shown to increase upon injury in oligodendrocyte lineage cells. Growth factors *Ccnf, Vgf,* and *Fgf2* were also upregulated, while synaptic transmission-related transcripts, particularly those involved in potassium and glutamate homeostasis, were downregulated in oligodendrocyte_1 subcluster | Permanent middle cerebral artery occlusion model in rats and thromboembolic stroke model in mice | Rat and mice | NovaSeq 6000 |
| Oligodendrocyte | *Serpina3n, C4b, Apoe, Trem2* | Following cerebral ischemia, reactive oligodendrocyte subclusters expressing *Serpina3n* or *C4b* markers, with neuroinflammation-mitigating potential, have been identified. The lesion core-periphery boundary accumulates chemokine, *Apoe, Trem2* co-expression, with marked reactive glial signature upregulation | Permanent middle cerebral  artery occlusion and 1, 3, and 7 d following injury | Mice | 10×Genomics |
| Oligodendrocyte | *Sult1a1* | Under 4-vessel occlusion in the hippocampal CA1 region, oligodendrocytes are grouped into 4 clusters, with oligodendrocyte subcluster 4 as immature ones expressing *Sult1a1* | Four vessel occlusion surgery | Rat | 10×Genomics |
| Astrocyte | *Cyr61, Fos, Cdkn1a, Jund, Fkbp5, Phkg1, Hes5, Sox2, Gm3764, Hbb-bs* | Compared with the sham group, genes such as *Cyr61, Fos, Cdkn1a, Jund,* and *Fkbp5* were upregulated in the MCAO group, while *Phkg1, Hes5, Sox2, Gm3764,* and *Hbb-bs* were downregulated | MCAO 1 h and reperfusion 24 h | Mice | 10×Genomics |
| Astrocyte | *Itm2a, Dbp, Hist2h2aa1, Spock2, Rps27rt,* *Spp1, Ccl4, Gfap, Cd14, Ccl12* | In sham samples, astrocyte_B exhibited a higher cellular proportion compared to astrocyte_C, whereas MCAO samples displayed the reverse trend. Astrocytes evolved from a dormant state (astrocyte_A) into two distinct functional states: astrocyte_B subcluster (*Itm2a, Dbp, Hist2h2aa1, Spock2,* and *Rps27rt*), and astrocyte_C subcluster (*Spp1, Ccl4, Gfap, Cd14,* and *Ccl12*). The volcano plot revealed a notable upregulation of genes in astrocyte_C and a concurrent downregulation of genes in astrocyte_B | MCAO 2 h and reperfusion 12 h | Mice | 10×Genomics |
| Astrocyte | *Apoe, Fabp5, Cd81, Fabp5, Aldoc* | *Apoe, Fabp5, Cd81,* and *Fabp5* are astrocyte-associated genes in the infarct site. RNAScope confirmed consistent *Aldoc* expression in astrocytes across injured and uninjured conditions, supporting its role as a reliable pan-astrocyte marker | Endothelin 1 (ET-1) stroke | Mice | 10×  Chromium) |
| Astrocyte | *Hmgb1, Vegfa, Camk2n1* | Compared to the sham group, a subcluster of astrocytes after low-intensity focused ultrasound stimulation showed upregulated *Hmgb1* and *Vegfa*, reduced microvessel count and cerebral blood flow, decreased *Camk2n1* expression, elevated extracellular calcium and glutamatergic synapses, increased dendritic spine density, and improved neurobehavioral impairments | MCAO 1.5 h and reperfusion 24 h | Mice | 10×Genomics |
| Astrocyte | *Nes*, *Ascl1* | 4 distinct astrocyte subclusters were identified after cerebral ischemia-reperfusion, *Nes* and *Ascl1* in astrocyte_2, demonstrate robust expression after MCAO, while showing almost negligible expression in the astrocyte_1, thereby pointing to a neurotrophic role | MCAO 1 h and reperfusion 12 h | Mice | Illumina HiSeq X platform |
| Astrocyte | *S100b*, *Aldoc, Nrxn1, Nlgn1* | Under 4-vessel occlusion in the hippocampal CA1 and CA3-DG, the astrocyte subcluster 1, rich in *S100b* and *Aldoc*, is linked to angiogenesis and vascular development, while the astrocyte subcluster 2, with high *Nrxn1* and *Nlgn1* expression, is associated with synaptic assembly and organization | Four vessel occlusion surgery | Rat | 10×Genomics |
| Dendritic cell | *H2-Aa, H2-Ab1, CD74* | Highly express MHC class II genes (*H2-Aa, H2-Ab1, CD74*) expressed in dendritic cells infiltrate the brain during acute/subacute phases | MCAO 6 h and reperfusion 1 d, 3 d, and 7 d | Mice | 10×Genomics |
| T cell | *Foxp3* | Following a stroke, there is a substantial buildup of *Foxp3^+^* Treg cells in densely populated brain regions | MCAO 1 h and reperfusion 1-5 week | Mice | 10×Genomics |
| Monocyte | *Ctss* | Cathepsin S (*Ctss*) degraded BBB junctional adhesion molecule proteins, disrupting the BBB. *Ctss* peaked in monocytes 3 days post-MCAO. *Ctss* knockout reduced infarct size, improved neurological scores, and lowered apoptosis/vascular leakage | MCAO 90 min and reperfusion 0, 1, 3, 7, and 14 d | Mice | 10×Genomics |
| Neutrophil | *Cxcl1, Cd63, Ptafr, Hcar2, Ifn, Ifitm1, Gbp2, Isg15, Irf7, Stfa2l1, Cxcr2, Ltb4r1, Trem1, Fpr1, Ccr1, Ltf, Camp, Cybb, Cd177* | Neutrophil_0 subcluster significantly expressed much higher *Cxcl1, Cd63, Ptafr* and *Hcar2* corresponding to the neutrophil mediated immunity. Neutrophil_1 subcluster featured higher expression of type I interferon (*Ifn*), *Ifitm1, Gbp2, Isg15, and Irf7*. Neutrophil_2 subcluster highly expressed genes response to cytokine stimulus *Stfa2l1, Cxcr2, Ltb4r1, Trem1, Fpr1,* and *Ccr1*. A list of secondary granules associated genes previous labeled preNeu and imNeu were identified mostly within neutrophil_3 subcluster, such as *Ltf, Camp, Cybb, Cd177* | MCAO 24 h | Mice | 10×Genomics |
| Neutrophil | *CD101+, CD62L^lo^ mature, CD177^hi^, CD101lo, CD62L^lo^, CD177^lo^, CD101^lo^, CD62L^hi^, Cxcl3* | Aged mice exhibit an amplified granulopoietic response post-stroke, resulting in blood accumulation of atypical neutrophils (*CD101^+^, CD62L^lo^ mature, CD177^hi^, CD101lo, CD62L^lo^, CD177^lo^, CD101^lo^, CD62L^hi^* immature) with heightened oxidative stress, phagocytosis, and procoagulant traits. *CD62L^lo^* neutrophils in aged mice produce *Cxcl3*, crucial for aging-associated neutrophil development and pathogenicity | **MCAO 45 min** | **Mice** | 10×Genomics |
| Neutrophil | *Aif1, Ly86, Il1b, ltf, Camp, Ngp* | Neutrophil_1 subcluster (*Aif1, Ly86, Il1b*) likely arises from infiltrated, mature neutrophils post-stroke, whereas Neutrophil_2 (*ltf, Camp, Ngp*) represents immature neutrophils maintaining homeostasis | MCAO 6 h and reperfusion 1, 3, and 7 d | Mice | 10×Genomics |
| Neuron | *Mt1, Mt2, Gfap, Ccl4, Ay036118, Gm17750, Nr2f1, Stmn2, Basp1, Cd24a* | In contrast to the sham group, *Mt1, Mt2, Gfap, Ccl4,* and *Ay036118* were highly expressed in the neurons of MCAO group, while *Gm17750, Nr2f1, Stmn2, Basp1,* and *Cd24a* were reduced | MCAO 1 h and reperfusion 24 h | Mice | 10×Genomics |
| Neuron | *Dnaja1, Hsp90aa1, Hspa8, Hsph1, Adarb2, Satb2* | A canonical stress response emerged in the ambiguous GABAergic neuronal cluster, marked by upregulation of heat shock proteins (*Dnaja1, Hsp90aa1, Hspa8, Hsph1*). *Adarb2* includes glutamatergic interneurons, as well as *Satb2* includes in glutamatergic neurons | Permanent middle cerebral artery occlusion in rats and thromboembolic stroke model in mice | Rat and mice | NovaSeq 6000 |
| Neuron | *Atf4* | Compared with the MCAO/R group, the intervention group with extracellular vesicles delivering quercetin-3-O-β-D-glucuronic acid could regulate neuronal *Atf4* to exert anti-inflammatory and neuroprotective effects | A microvascular clip transiently clamped the right common carotid artery for 2 h to induce cerebral ischemia, then removed to restore circulation for 24 h reperfusion | Mice | 10×Genomics |
| Endothelial cell | *Ifit3, Isg15, Usp18, Spp1, Cxcl2, Ccl4, Cd14* | Two endothelial cell clusters were found capillary and arterial (*Ifit3, Isg15, Usp18*) though their proportion declined in the MCAO group. Ischemia altered inflammatory cytokine gene expression (*Spp1, Cxcl2, Ccl4, Cd14*) in each endothelial cell subclusters | MCAO 24 h | Mice | 10×Genomics |
| Endothelial cell | *Cldn5*, *Flt1* | Endothelial_0 subcluster (account for ~90% of young sham group cells) expressing *Cldn5* and *Flt1* was designated as homeostatic endothelial cells predominating in nonischemic conditions | MCAO 24 h and reperfusion 3 d and 14 d | Mice | 10×Genomics |
| Endothelial cell | *Tmem252, Akap12, Mt1, Mt2, Lcn2, Hmcn1, Tfrc, Car4, Slc16a1, Tgfb2* | In endothelial cells, the MCAO group exhibited upregulation of genes (*Tmem252, Akap12, Mt1, Mt2, Lcn2*) and downregulation of genes (*Hmcn1, Tfrc, Car4, Slc16a1, Tgfb2*) | MCAO 1 h and reperfusion 24 h | Mice | 10×Genomics |
| Endothelial cell | *Ackr1, Ecscr, yms, Dut, Dctpp1, Tyms, Dut, Dctpp1, Plvap, Plpp3, Igfbp3, Plpp1, Cd24a, Ldb2, Igf1r* | Lrg1 activates endothelial cells post-stroke, revealing endothelial_9 subcluster. Endothelial_6 expresses venular marker *Ackr1*; Endothelial_2 (*Ecscr*) indicates a reactive state for migration/vessel formation. Endothelial _9 shows metabolic (*Tyms, Dut, Dctpp1*) and proliferation (*Pimreg, Pclaf*) genes. Endothelial_8 has fenestrated brain endothelial traits (*Plvap, Plpp3, Igfbp3, Plpp1, Cd24a, Ldb2*). Endothelial_1, 3, and 5 elevate *Igf1r* expression at 2 d and 14 d post-stroke | MCAO 2 h and reperfusion 12 h | Mice | 10×Genomics |
| Endothelial cell | *Ndufa1, Sdhb, Uqcr11, Cox5b, Cox7b, Mdh1* | In MCAO/R group, there was an elevated proportion of endothelial cells cluster 3, accompanied by the upregulated expression of genes implicated in the oxidative phosphorylation pathway (including *Ndufa1, Sdhb, Uqcr11, Cox5b, Cox7b,* and *Mdh1*) | MCAO 1.5 h and reperfusion 24 h | Mice | 10×Genomics |
| Pericyte | *Cd74* | Pericytes in the CA3-DG region of hippocampus after 4-vessel occlusion showed significantly higher *Cd74* expression than in the sham-operation group | Four vessel occlusion surgery | Rat | 10×Genomics |
| Vascular mural cells or pericyte or rouget cell | *Car4, Art3, Acta2* | Vascular smooth muscle cells_5 subcluster (*Car4, Art3*), with a reduced cell proportion in the MCAO group, aligning with prior studies and linked to the type I interferon signaling pathway. Pericyte_3 subcluster, marked by high *Acta2* expression for cerebral blood flow regulation, are associated with pericyte differentiation potential | MCAO 24 h | Mice | 10×Genomics |
| Perivascular fibroblast-like cell | *Slc22a6,Slc6a13, Slc7a11, Slc1a3, Col1a1, Col1a2, Col12a1,Vcam1, Lum, Dcn, Ifitm1, Ifitm2, Gsn, Fxyd5, Atp1b1, Slc38a2,*  *Slc16a11, Slc6a6, Slc26a2,*  *Slc4a10* | Perivascular fibroblast-like cell_0 subcluster specifically expressed some genes related to membrane transporters (*Slc22a6, Slc6a13, Slc7a11, and Slc1a3*) and collagen fibril organization genes (*Col1a1, Col1a2, Col12a1*). Perivascular fibroblast-like cell_1 subcluster was enriched in the process of extracellular matrix organization (*Vcam1, Lum, Dcn*) and response to interferon-beta (*Ifitm1, Ifitm2, Gsn*). Perivascular fibroblast-like cell_2 subcluster highly expressed genes encoding pumps (*Fxyd5* and *Atp1b1*), carboxylic acid transport (*Slc38a2, Slc16a11, Slc6a6*), and regulation of cellular pH (*Slc26a2, Slc4a10*) | MCAO 24 h | Mice | 10×Genomics |
| Myeloid cell | *Apoe, Lyz2, Tmem119, Hexb, Cx3cr1, P2ry12, Cst3, Sparc, Spp1, Fabp5, Gpnmb, Lpl, Mmp12, Csf1, Adam8* | Stroke-associated myeloid cells display a mixed macrophage-microglial phenotype, sharing similarities with macrophage clusters (*Apoe, Lyz2*) and canonical microglia genes (*Tmem119, Hexb, Cx3cr1, P2ry12, Cst3, Sparc*). Stroke-associated myeloid cells uniquely express high levels of *Spp1, Fabp5, Gpnmb, Lpl, Mmp12, Csf1,* and *Adam8*. At 72 h post-ischemia, Stroke-associated myeloid cells show slight expression changes, upregulating macrophage-associated genes (*Apoe, Lyz2*) along with *Fabp5* and *Gpnmb*, suggesting they are specialized, activated myeloid cells with high lysosomal and phagocytic activity, akin to embryonic microglia in clearing lipid debris | MCAO 30 and 45 min and two post-ischemia  times 24 and 72 h | Mice | 10×Genomics |
